# The chromosome-level genome of *Eucommia ulmoides* provides insights into sex differentiation and α-linolenic acid biosynthesis

**DOI:** 10.3389/fpls.2023.1118363

**Published:** 2023-03-31

**Authors:** Qingxin Du, Zixian Wu, Panfeng Liu, Jun Qing, Feng He, Lanying Du, Zhiqiang Sun, Lili Zhu, Hongchu Zheng, Zongyi Sun, Long Yang, Lu Wang, Hongyan Du

**Affiliations:** ^1^Research Institute of Non-timber Forestry, Chinese Academy of Forestry, Zhengzhou, China; ^2^Key Laboratory of Non-timber Forest Germplasm Enhancement and Utilization of National Forestry and Grassland Administration, Chinese Academy of Forestry, Zhengzhou, China; ^3^Engineering Research Center of Eucommia ulmoides, State Forestry and Grassland Administration, Zhengzhou, China; ^4^Agricultural Big-Data Research Center and College of Plant Protection, Shandong Agricultural University, Taian, China; ^5^Academy of Chinese Medical Sciences, Henan University of Chinese Medicine, Zhengzhou, China; ^6^Product Department, Henan Jinduzhong Agricultural Science and Technology Co., Ltd., Yanling, China; ^7^Operation Department, Grandomics Biosciences Co., Ltd., Wuhan, China

**Keywords:** *Eucommia ulmoides* Oliver, genome, whole-genome duplication, MADS-box genes, sex differentiation, α-linolenic acid biosynthesis

## Abstract

*Eucommia ulmoides* Oliver is a typical dioecious plant endemic to China that has great medicinal and economic value. Here, we report a high-quality chromosome-level female genome of *E. ulmoides* obtained by PacBio and Hi-C technologies. The size of the female genome assembly was 1.01 Gb with 17 pseudochromosomes and 31,665 protein coding genes. In addition, Hi-C technology was used to reassemble the male genome released in 2018. The reassembled male genome was 1.24 Gb with the superscaffold N50 (48.30 Mb), which was increased 25.69 times, and the number of predicted genes increased by 11,266. Genome evolution analysis indicated that *E. ulmoides* has undergone two whole-genome duplication events before the divergence of female and male, including core eudicot γ whole-genome triplication event (γ-WGT) and a recent whole genome duplication (WGD) at approximately 27.3 million years ago (Mya). Based on transcriptome analysis, *EuAP3* and *EuAG* may be the key genes involved in regulating the sex differentiation of *E. ulmoides*. Pathway analysis showed that the high expression of ω-3 fatty acid desaturase coding gene *EU0103017* was an important reason for the high α-linolenic acid content in *E. ulmoides*. The genome of female and male *E. ulmoides* presented here is a valuable resource for the molecular biological study of sex differentiation of *E. ulmoides* and also will provide assistance for the breeding of superior varieties.

## Introduction

There has been a wide range of uses for *Eucommia ulmoides* Oliver, such as medicinal and natural rubber extraction purposes. Because there has been a long history of application of this plant in China (Zhu and Sun, 2018), it has aroused the interest of many researchers. In 2018, the first draft reference genome of *E. ulmoides* (Male V1) was released based on next-generation sequencing, and consisted of a total genome length of 1.18 Gb genome with contig N50 that was 17.06 kb in length, and the assembled genome size was larger than the 1.1 Gb predicted by 17-mer analysis. A large number of superscaffolds were assembled from contigs, but none was completely anchored to the chromosomes (Wuyun et al., 2018).

It was realized that the accuracy and completeness of this genome assembly could be further refined. A new version of the genome was subsequently published in 2020, whereby the authors first obtained the *Eucommia* haploid plant through parthenogenesis, and then sequenced and assembled it. The genome size was 947.86 Mb, accounting for 92.93% of the estimated genome size (1.02 Gb). Contig N50 was 13.16 Mb, and scaffold N50 was 53.15 Mb in length, and long scaffolds were further anchored to 17 pseudochromosomes by Hi-C (Li et al., 2020). Although two versions of the *E. ulmoides* genome have been released, a high-quality female *E. ulmoides* genome has yet to be produced, which is necessary for the study of evolution and sex differentiation.

**Figure 1:**
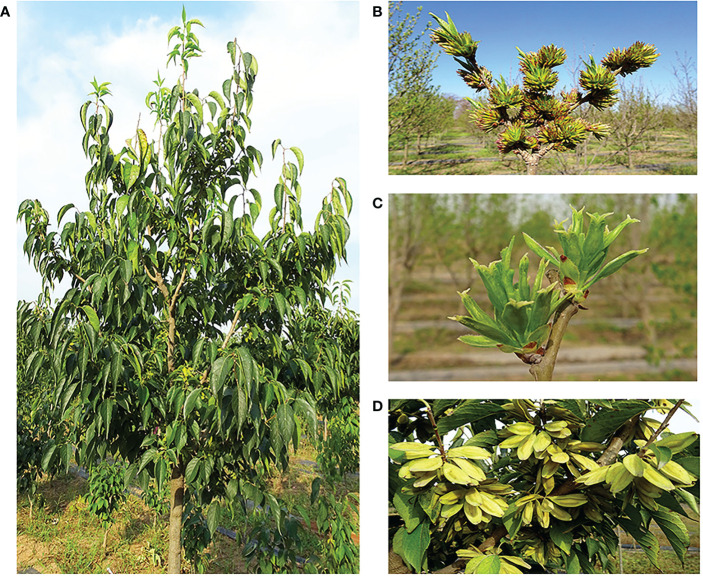
Morphological characteristic of *Eucommia ulmoides*. **(A)** Mature plant, **(B)** male inflorescence, **(C)** female inflorescence, **(D)** fruit.

*E. ulmoides* is a dioecious perennial woody plant, with male and female inflorescences that greatly vary, and thus, the quantities of important compounds in male and female plant are different (**Figure 1**). The utilization value of *E. ulmoides* also varies with the sex of the plant (Wang et al., 2020). In flowering plants, the sex difference is mainly manifested as different flower organs, and therefore, genes related to flower development may participate in the process of sex differentiation (Barrett and Hough, 2012; Massonnet et al., 2020). As the key regulators of almost all aspects of plant reproductive development, MADS-box genes play a particularly prominent role in flowering time control, inflorescence structure, floral organ identity determination, and seed development. However, the floral induction and floral organ regulation genes and regulatory networks of *E. ulmoides* are still unclear.

Based on the studies of model plants *Arabidopsis thaliana* (Bowman et al., 1989) and *Antirrhinum majus* (Schwarz-Sommer et al., 1990), the genetic regulation mechanisms that govern flower organ development have been preliminarily explored, and the famous ABC model of flower organ development has been proposed (Coen and Meyerowitz, 1991; Soltis et al., 2007), which was later supplemented by the ABCDE model (Theissen, 2001; Causier et al., 2010). Except for the *AP2* gene, all the genes involved in the ABCDE model were MADS-box genes belonging to different functional categories (Hu et al., 2021). Therefore, the MADS-box gene family may play an important role in regulating the sexual differentiation of flowers, and a large number of MADS-box genes and their functions have been found and verified in plants such as *Arabidopsis* (Di Marzo et al., 2020), *Orchidaceae* (Teo et al., 2019), *Petunia* (Morel et al., 2019), *Oryza sativa* (Yin et al., 2019) and *Zea mays* (Abraham-Juárez et al., 2020). A previous study based on comparative transcriptome analysis of male and female *E. ulmoides* identified putative sex-associated genes (Wang and Zhang, 2017). Regrettably, only one gene with high homology to the *AP3* gene in *Arabidopsis* was detected, which was a B class organ identity gene. Although that study provides a reference for exploring the sex differentiation of *E. ulmoides*, there is currently a lack of information on the sex differentiation mechanism of *E. ulmoides*.

In plants, stearic acid is catalyzed by fatty acid desaturase to produce oleic acid and linoleic acid, and eventually α-linolenic acid (Li-Beisson et al., 2013), which is an essential fatty acid and is very important for human health (Kim et al., 2014). There has been prior study on α-linolenic in plants such as *Perilla frutescens* (Liao et al., 2018), *Plukenetia volubilis* (Hu et al., 2018), *Paeonia ostia* (Yu et al., 2021). *E. ulmoides* is a woody plant with a very high α-linolenic acid content, even more than 60% in its seed oil (Zhang et al., 2018), well above purslane which has the highest content of linolenic acid in green leafy vegetables (30.15%). Although the synthesis of α-linolenic acid in *E. ulmoides* has also been studied from the glycolytic pathway based on transcriptome analysis (Feng et al., 2016), the information in genes encoding key enzymes for linolenic acid synthesis and the mechanism of α-linolenic acid accumulation are still not clear.

In this study, we aimed to construct a female and male *E. ulmoides* genome and explore the molecular mechanism of sex differentiation and α-linolenic acid accumulation in *E. ulmoides*. Our results will provide essential resources for studies on *E. ulmoides* evolution and directional variety improvement, and also lay a foundation for the rapid identification of male and female plants, thus improving the utilization rate of *E. ulmoides*.

## Materials and methods

### Plant samples

Fresh leaves for genome sequencing were collected from a female *E. ulmoides* plant ‘Huazhong No. 8’, which was used to assemble the Female V1 genome. To observe the morphological structure of female and male flower bud development, flower buds were collected from female ‘Huazhong No. 6’ and the male ‘Huazhong No. 11’ during the entire development period. Samples were obtained every 5 days from the beginning of bud germination in late April to the end of September, once every 10 days from early October to January of the following year, and once every 3 days from February to early April of the following year, with 20 female and male flower buds being collected each time. The experimental materials for sex differentiation analysis were derived from the collected flower buds. Male and female flower buds at the floral organ induction stage (the inflorescence primordium formation stage), floral organ morphological differentiation initial stage (the pistil and stamen differentiation stage), and flower organ maturity stage were selected for transcriptome sequencing, with three biological repetitions for each sample. To analyze the expression of α-linolenic acid synthesis genes, four different tissues (stem, bark, leaf and fruit) of female ‘Huazhong No. 8’ were extracted. All plant samples were collected from the Yuanyang Experimental Base of Research Insitute of Non-timber Forestry, Chinese Academy of Forestry. Plant materials were immediately immersed in liquid nitrogen, transported to the laboratory, and then stored in a -80°C freezer for sequencing.

### Morphological and structural observation of flower bud development

After collection, the materials were immediately immersed in FAA fixative solution (70% ethanol: glacial acetic acid: 38% formaldehyde =18:1:1), transported to the laboratory, and then stored in a -80° freezer. Materials preserved in FAA fixative solution were sliced using a conventional paraffin-sectioning method. Then the slices were observed and photographed using OLYMPUS optical microscope.

### Sequencing and library construction

Total genomic DNA was extracted from fresh leaves using the QIAGEN^®^ Genomic DNA extraction kit (Cat#13323, QIAGEN) for PacBio sequencing on a PacBio Sequel II instrument. For Hi-C library construction and sequencing, restriction endonucleases (HindIII/MboI) were used for chromatin digestion, and after biotin labeling, flat-end binding and DNA purification. Hi-C samples were prepared and sampled for DNA quality testing. The Hi-C fragment consisted of removed biotin that was fragmented by ultrasound, end-repaired, and after the addition of base A, the biotin-containing fragment was isolated and then sequenced to form the jointed product. Then, Polymerase Chain Reaction (PCR) conditions were tested, and DNA was amplified to obtain the library products (Kong and Zhang, 2019). An Illumina HiSeq X Ten sequencer was used for sequencing after the library quality control was confirmed.

RNA was extracted using a TRIzol kit (Invitrogen) according to the manufacturer’s instructions. The RNA concentration was measured using a NanoDrop spectrophotometer, and RNA purity and integrity were determined by an Agilent 2100 Bioanalyzer and 1% agarose gel electrophoresis, respectively. After passing the quality inspection, a cDNA library was constructed according to the instructions accompanying the cDNA Library Construction Kit (NEB). Paired-end sequencing was performed on the cDNA library using the Illumina HiSeq X-10 platform, and 150 bp paired-end sequences were generated.

### Genome assembly and evaluation

For the genome assembly of female *E. ulmoides* plant ‘Huazhong No. 8’, first, sub-sequences from PacBio sequencing were assembled using Falcon v2.0.5 (https://github.com/PacificBiosciences/FALCON/) with the parameter ‘–max diff 100 –max cov 100 –min cov 2 –min len 5000’, the pure third-generation assembly software officially launched by PacBio. Then, Arrow (Chin et al., 2013) was applied to align the third generation data to the preliminary assembly for correction with default parameters. After the initial genome assembly was completed, Pilon v1.22 (Walker et al., 2014) with the parameter ‘–mindepth 10 –changes –fix bases’ was used for iterative polishing. The Hi-C data were aligned to the genome assembly *via* Juicer v1.5 software with the following parameter settings: -s DpnII -t 20 (Durand et al., 2016b). Finally, the draft genome of *E. ulmoides* was assembled onto chromosomes by 3D-DNA pipeline with the parameter ‘-m haploid -s 4 -c 17 -j 15’ (Dudchenko et al., 2017). The contact matrix and heatmap of chromosomes were drawn with Juicebox (Durand et al., 2016a).

As for the male *E. ulmoides* tree (SNJ) genome reassembly, named Male V2 genome, we first downloaded the previous genome assembly data from the NCBI under accession number SRP095726. Next, the downloaded genome was refined using Hi-C data with the same method as that used for the female genome assembly. Benchmarking Universal Single-Copy Orthologs (BUSCO) v4.1.4 (https://busco.ezlab.org/) was used to assess the integrity and accuracy of the final genome assembly with the Eudicotyledons_odb10 database (Simão et al., 2015).

### Genome annotation

TRF v4.07 (Benson, 1999) (tandem repeats finder) software (https://tandem.bu.edu/trf/trf.html) was applied to predict tandem repeats of the genome. Transposable elements (TEs) were identified by combining *de novo*-based and homology-based approaches. In the *de novo*-based approach, RepeatModeler v1.0.11 (Flynn et al., 2020) (http://www.repeatmasker.org/RepeatModeler/) and LTR-FINDER v1.05 (Xu and Wang, 2007) were used to build the repeat library (LTR length 100 to 5000 nt; length between two LTRs: 1000 to 20,000 nt), and then, RepeatMasker v4.0.7 (Tarailo-Graovac and Chen, 2009) (http://www.repeatmasker.org/) was used to identify and classify repeats under the parameter ‘-a -e ncbi -q -norna -nolow -div 30 -cutoff 225’. For the homology-based approach, the known repetitive sequences database Repbase (Bao et al., 2015) (https://www.girinst.org/repbase/) was searched by RepeatMasker to find homologous sequences in the *E. ulmoides* genome.

Gene prediction for the *E. ulmoides* genome was performed by integrating three different methods: *de novo* prediction, homology-based prediction and transcriptome-based prediction. *De novo* gene prediction was achieved using Augustus v3.3 (parameter: -strand=both -genemodel=partial -gff3=on -species= arabidopsis) (Stanke and Morgenstern, 2005) (http://bioinf.uni-greifswald.de/augustus/), SNAP (Korf, 2004) and GlimmerHMM v3.52 (Majoros et al., 2004). For the homology approach, BLASTP (Camacho et al., 2009) was used to map protein sequences onto the *E. ulmoides* genome, and GeneWise v2.4.1 (Birney et al., 2004) was used to align the homologous genome sequences with the matching proteins. RNA-seq sequences were mapped to the genome assembly using PASA v2.0.2 to identify putative exon regions and splice junctions. Finally, EVidenceModeler v1.1.1 (Haas et al., 2008) (https://evidencemodeler.github.io/) was employed to integrate the gene sets predicted by the three methods. For gene annotation, EggNOG-mapper v4.5 (Huerta-Cepas et al., 2017) software was employed with emapper DB 4.5.1 to obtain the Clusters of Orthologous Genes (COG), eggNOG, Gene Ontology (GO) and KEGG pathway information for each gene. HMMER v3.2.1 (Prakash et al., 2017) was applied to search the Pfam database (http://pfam.xfam.org/) and obtain the protein domains. Functional annotation of protein coding genes was performed using the UniProt database (https://www.uniprot.org/).

Four types of non-coding RNAs (ncRNAs), tRNA, rRNA, miRNA, and snRNA, were annotated in the *E. ulmoides* genome. TRNAscan-SE v1.4 (Lowe and Chan, 2016) (http://lowelab.ucsc.edu/tRNAscan-SE/)software was employed to identify the tRNAs with tRNAscn-SE -i -q and eukaryote parameters. rRNAs were detected using BLASTN alignment of rRNA sequences known related species. Other ncRNAs were predicted using INFERNAL v1.1 (Nawrocki et al., 2009) software with the parameter ‘cmsearch –ga –incE 0.01 -E 10.0’ to search the Rfam v12.0 (Nawrocki et al., 2015) database (http://rfam.xfam.org/).

### Detection of WGD events

To identify whole genome duplication (WGD) events in *E. ulmoides*, BLASTP was used to perform a homology search using the female and male genome, and MCScanX v0.8 (Wang et al., 2012) (https://sourceforge.net/projects/mcscanx/) was employed to detect syntenic blocks. The collinearity analysis of male and female was conducted by JCVI v1.2.1. Ks (synonymous substitution) values for paralogous blocks were calculated by KaKs_Calculator v2.0 with the parameter ‘-i test.axt -m MA -c -o’ (Wang et al., 2010), and a frequency distribution graph of the Ks values was drawn to identify WGD events in *E. ulmoides*. The WGD event time was estimated by the formula ks/2r, with a r value of 8.25 e-9.

### MADS-box genes analysis

To identify MADS-box genes, HMMER v3.2.1 software with the SRF-TF domain (PF00319) and K-box (PF01486) were used to search against the *E. ulmoides* genome proteins (–cut_ga, E value<1×10^−5^), which were obtained from Pfam. The search results containing MADS-box domain or K-box domain were retained, and incomplete functional domain sequences were removed. Using all Arabidopsis MADS-box genes as queries, the predicted *E. ulmoides* MADS genes were checked by BLASTP searches (E value<1×10^−5^). The phylogenetic tree was then constructed using RAxML v8.2.12 with the GTRGAMMA substitution model and 1000 bootstraps on the CIPRES website (https://www.phylo.org/portal2/home.action). The phylogenetic tree was visualized using FigTree v1.4.4. The combination of genome annotation, homology search and MADS-box gene expression in male and female flower buds, enabled the ABCDE model genes to be identified. Using all female *E. ulmoides* MADS-box genes as queries, BLASTP was used to map protein sequences onto the male genome. For these protein sequences, the phylogenetic tree was constructed through MEGA-X using the maximum likelihood method and the JTT matrix-based model with 1,000 bootstraps. Multiple sequence alignments were performed by CLUSTALW program and viewed in GeneDoc. The gene structure of these MADS-box genes was demonstrated by TBtools. The data of *Nymphaea colorata* was downloaded from BIG Data Center with the accession number GWHAAYW00000000. And the phylogenetic trees of genes related to sex differentiation in *E. ulmoides* and *N. colorata* were constructed using MEGA7 with the Neighbor Joining method.

### Identification of α-linolenic acid biosynthesis and metabolism genes

The KEGG database (https://www.kegg.jp/kegg/kegg1.html) was queried to identify genes involved in α-linolenic acid synthesis and metabolism. First, known genes related to α-linolenic acid biosynthesis and metabolic pathways in KEGG were selected and their protein sequences were downloaded. The downloaded protein sequences were aligned with the *E. ulmoides* genome using the NCBI online comparison function BLASTP (https://blast.ncbi.nlm.nih.gov/Blast.cgi), and the annotation information was combined to predict genes involved in α-linolenic acid synthesis and metabolism.

### Transcription analysis

FastQC v0.11.9 was used to estimate the quality of RNA sequences. To obtain clean sequences, Trimmomatic v0.36 was used to remove adapter sequences and low-quality sequences (with a base quality value less than 30 or greater than 5% unknown bases). To estimate the gene expression level, clean sequences from RNA-seq were mapped onto the final Female V1 genome assembly using Tophat2 v2.1.1 with the parameters: –b2-sensitive -N 2 -p 6 -g 10 –read-edit-dist 2 –read-mismatches 3 –read-gap-length 2 (Kim et al., 2013), and then fragments per kilobase per million (FPKM) of each gene was performed by Cufflinks v2.2.1 with the following parameter: -p 6 -g file.gff -u –library-type fr-unstranded (Trapnell et al., 2010) (http://cole-trapnell-lab.github.io/cufflinks/). Finally, the values of log_2_(FPKM+1)-that represented the gene expression level and the differentially expressed genes (DEGs) were identified using Cuffdiff v2.2.1, with a p-value (FDR) < 0.05 and |log2 (fold change)| > 1.

## Results

### Morphological and structural observation of female and male flower bud differentiation

**Figure 2:**
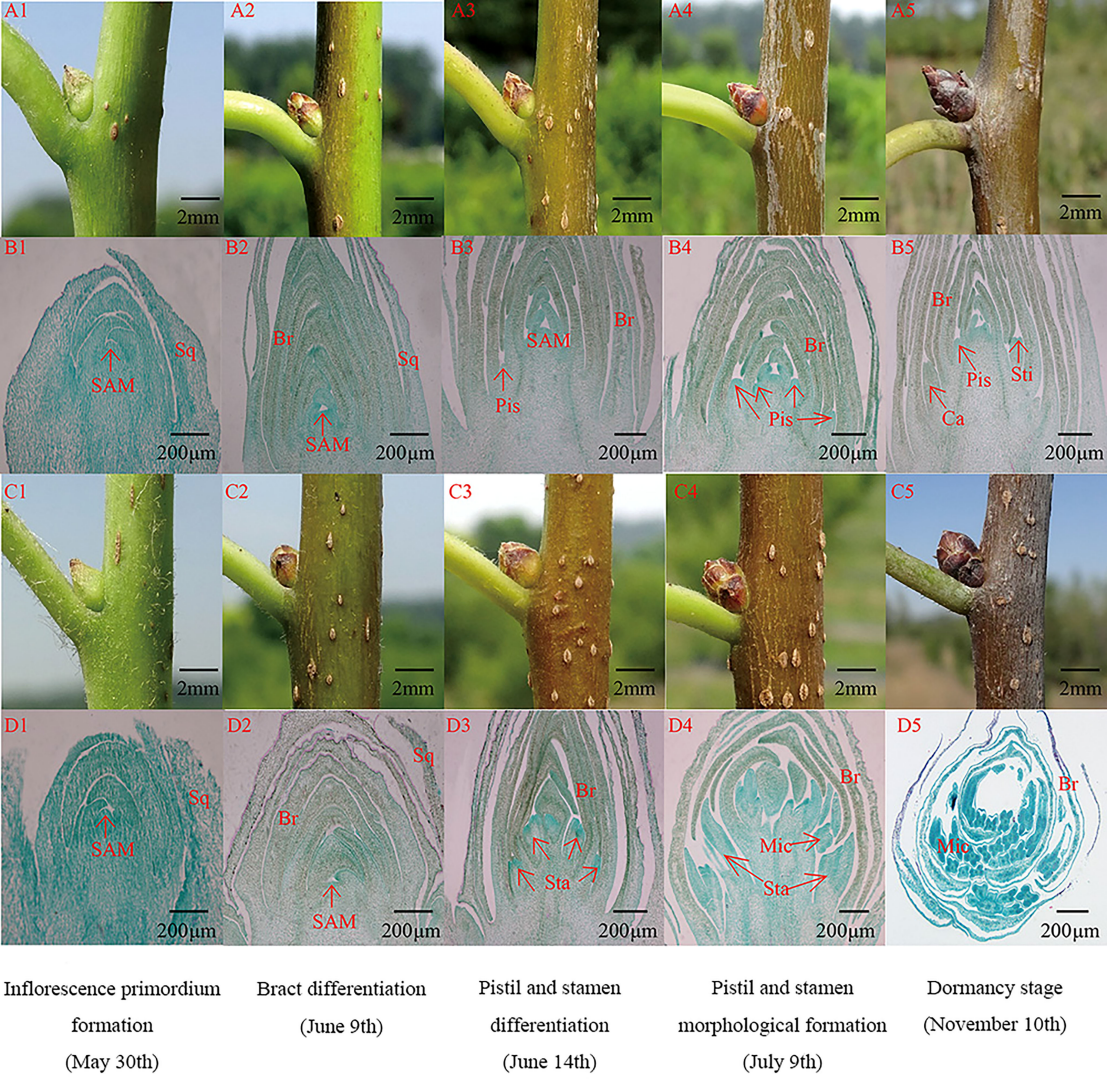
Internal anatomical structure and external morphological characteristics of male and female flower buds at different differentiation stages. Br, Bract; Ca, Carp; Mic, Microsporocyte; Pis, Pistil, SAM, Stem Apical Meristem; Sq, Squama; Sta, Stamen; Sti, Stigma. **(A1-A5)**: The external morphology structure of female flower bud; **(B1-B5)**: The anatomic structure of female flower bud; **(C1-C5)**: The external morphology structure of male flower bud; **(D1-D5)**: The anatomic structure of male flower bud.

With the growth and development of female and male flower buds of *E. ulmoides*, the cells of the flower bud meristem continued dividing, and the internal and external morphological structure gradually changed. The floral organ development of the female and male flower buds of *E. ulmoides* can be divided into four stages: inflorescence primordium formation, bract differentiation, pistil and stamen differentiation, and pistil and stamen morphological formation (**Figure 2**).

In the inflorescence primordium formation stage, the female and male flower buds exhibit similar morphological characteristics. The buds are young and green, approximately triangular, with white fluff on the surface (**Figures 2A1, C1**). The internal anatomical structure shows that the flower buds are apical and conical, with thick cytoplasm (**Figures 2B1, D1**).

In the bract differentiation stage, the female and male flower bud morphological characteristics remain relatively similar, and males and females cannot be accurately distinguished. The outer bracts of female and male flower buds gradually lignify into scales, and the color of the flower buds gradually changes from green to light brown (**Figures 2A2, C2**). The number of bracts increases, and the meristem at the bud apex widens and flattens (**Figures 2B2, D2**).

In the pistil and stamen differentiation stage, the internal structure of female and male flower buds showed obvious morphological differences. Round small protuberances form between the axils of the primordia of the female buds, namely the pistil primordia, and several stamen primordia that form between the axils of the male buds are clustered between the bracts. The male and female buds can be distinguished according to the sectioning results (**Figures 2B3, D3**).

In the pistil and stamen morphological formation stage, female flower buds grow faster longitudinally and are approximately conical in shape, while the stamens are approximately spherical (**Figures 2A4, C4**). A single pistil primordium is differentiated from the base to the center on both sides of the female flower bud, while the male flower bud is differentiated from several stamens to form a stamen cluster inside the bract (**Figures 2B4, D4**). The pistil shape is pinnate, with a central depression at the top that forms two protuberances, namely the pistil stigma. After the pistil completes its morphogenesis, and stamen pollen sac differentiates into the obvious endothecium, middle layer, and tapetum (**Figures 2B5, D5**). Then, the internal and external morphology of the flower bud does not change, and the bud enters the dormancy stage.

### Genome assembly and assessment

**Figure 3:**
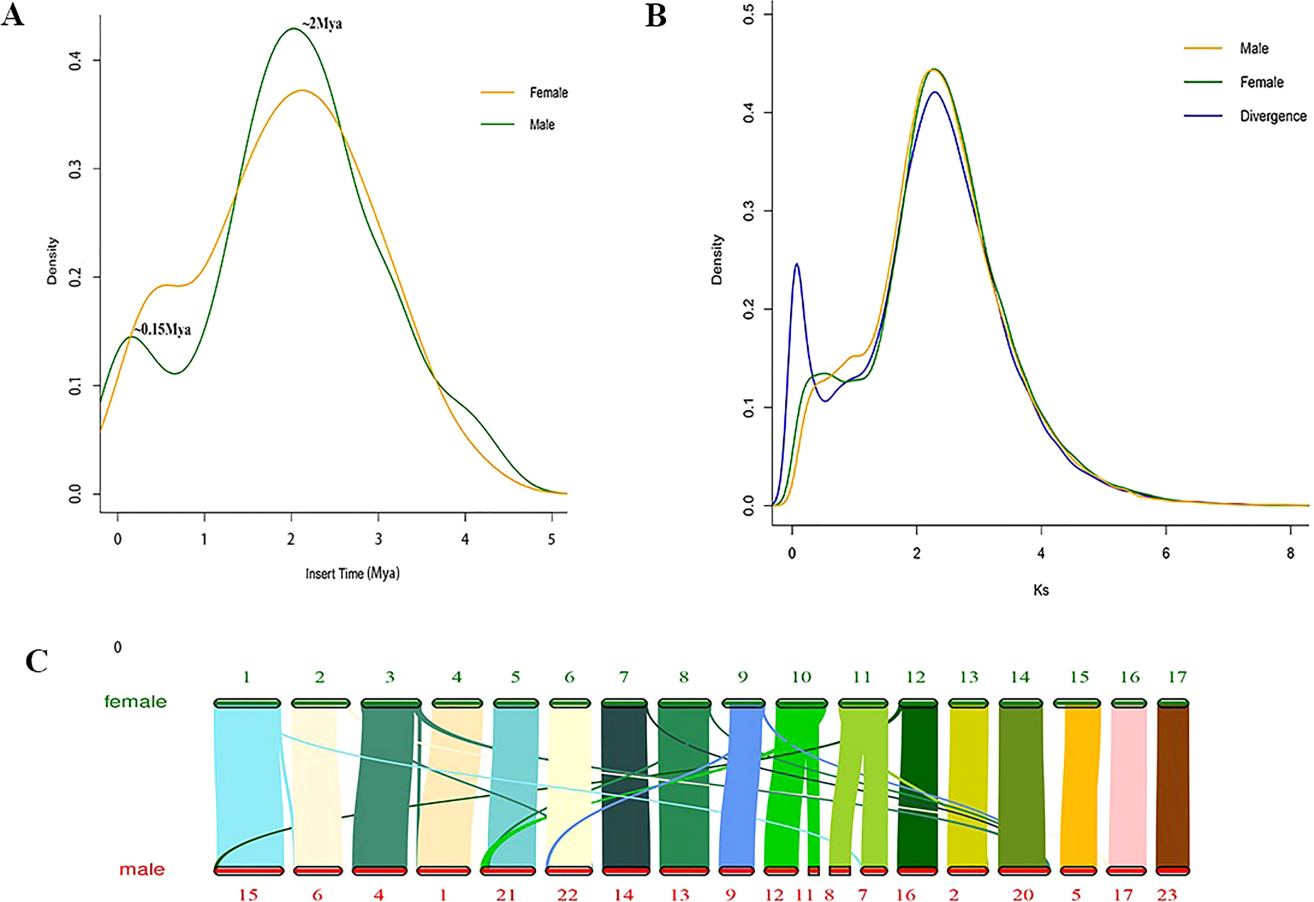
Genome evolutionary analysis. **(A)** Distribution of insertion times for LTR-RTs. **(B)** Ks value distribution in Female V1 and Male V2 genome. The divergence line represents Ks value distribution of syntenic blocks between male and female. **(C)** Genomic alignments of chromosomes and superscaffolds between Female V1 and Male V2.

A combined strategy of PacBio and Hi-C sequencing technologies was used to construct the high-quality female *E. ulmoides* genome assembly (Female V1). The PacBio long sequences were corrected and assembled into the preliminary genome assembly using FALCON. The obtained genome size was 957 Mb, which consisted of 2,662 contigs with contig N50 that was 1.3 Mb in length. Finally, with the assistance of 184.7 Gb of Hi-C sequences, the assembled contigs were anchored to 17 pseudochromosomes of 1.01 Gb with superscaffold N50 that was 51.89 Mb in length, and the GC content of the genome was 35.14% (**Table 1**). The interactions of contigs on pseudochromosomes were detected to adjust their order and direction in the Hi-C heat map, which also indicated the high quality of Female V1 genome assembly (**Supplementary Figures 1, 2**). The Male V2 genome was re-constructed using Hi-C technology. The genome size of the new version (Male V2) was 1.24 Gb, with 23 superscaffolds and the superscaffold N50 that was 48.30 Mb long, and the GC content was 35.19% (**Table 1**). Genomic alignments of chromosomes and superscaffolds between Female V1 and Male V2 are showed in (**Figure 3C**).

**Table 1:** Summary of the *Eucommia ulmoides* genome.

Type	Female V1	Male V2	Male V1
Genome Size	1.01 Gb	1.24 Gb	1.18 Gb
Contig N50	1.33 Mb	17.06 Kb	17.06 Kb
Scaffold N50	5.31 Mb	1.03 Mb	1.03 Mb
SuperScaffold N50	51.89 Mb	48.30 Mb	1.88 Mb
GC content	35.14%	35.19%	–
Complete BUSCOs	93.2%	92.1%	90%
Protein coding genes	31,665	37,998	26,732
Mean gene length	6,273 bp	6,199 bp	–
Mean coding sequence length	1,086 bp	1,007 bp	1,001
Mean number of exons per gene	5.18	4.45	4.74
Mean exon length	209 bp	226 bp	211 bp
Total repetitive sequence	68.26%	62.25%	61.24%
Total non-coding RNAs	2,488	2,865	3,201

To assess the quality of the two newly assembled genomes, BUSCO was carried out. Among the 2,121 plant-specific orthologs, 2,024 (95.4%) and 2,026 (95.5%) BUSCOs were identified in the Female V1 and Male V2 assembly respectively. In addition, 1,975 (93.2%, Female V1) and 1,954 (92.1%, Male V2) of the identified orthologs in the two genomes were considered to be complete, which were higher than the 90.0% completed genes identified in the Male V1 genome (**Supplementary Figure 3** and **Supplementary Table 1**). The BUSCO results suggested that both genomes were assembled with high quality.

### Genome annotation

**Figure 4:**
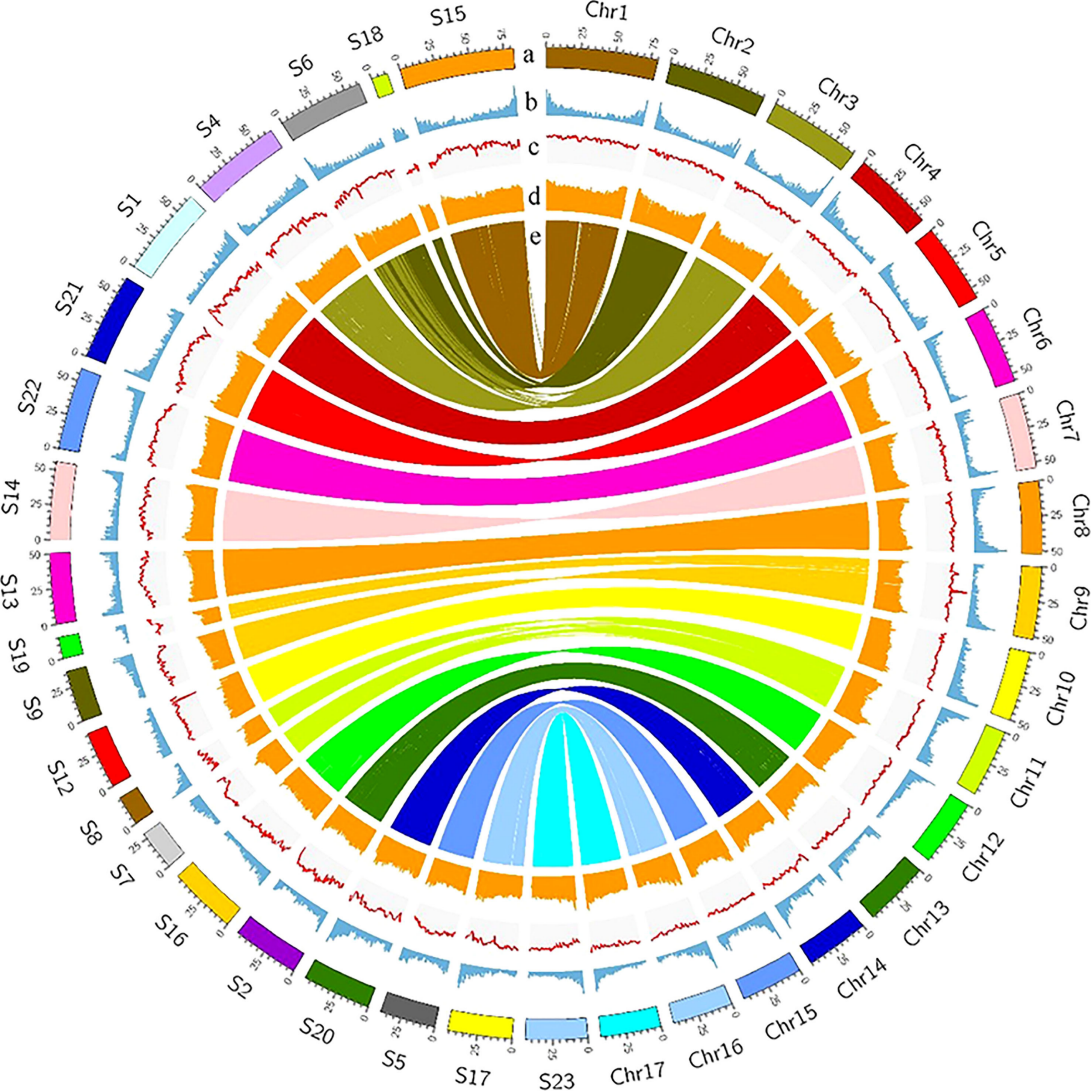
Landscape of Female V1 and Male V2 genome. The circle from outside to inside represents, **(A)** Chromosomes of Female V1 and Superscaffolds of Male V2, **(B)** gene density, **(C)** GC content, **(D)** repeat abundance, **(E)** synteny information. All distributions were drawn in a window size of 1 Mb.

The Female V1 and Male V2 genome consisted of 68.26% (688.75 Mb) and 62.25% (770.88 Mb) repeats, respectively (**Table 1** and **Figure 4**). In both genomes, LTR (long terminal repeat) was the most abundant type of repetitive element, accounting for 40.15% (405.08 Mb) and 36.6% (453.30 Mb) of the genome size, respectively. Additionally, 59.05 Mb (5.85% of the Female V1 genome) and 64.56 Mb (5.21% of the Male V2 genome) DNA transposons were identified, which were the second highest repeated sequence in both genomes (**Supplementary Table 2**). The LTR-RT insertion time was estimated to provide a retrospective view of the expanded history of LTR-RTs in the two newly assembly *E. ulmoides* genomes. LTR-RTs gradually accumulated over 5 million years ago, and there was an insertion explosion approximately 2 million years ago in both genomes, with the Male V2 genome undergoing another peak at 0.15 Mya (**Figure 3A**).

A combination of ab initio prediction, RNA-seq, and homology-based search was used to predict the protein-coding genes in the *E. ulmoides* genome. Overall, 31,665 and 37,998 genes were predicted in the Female V1 and Male V2 genome, and the average protein-coding gene size was 6,273 bp and 6,199 bp respectively (**Table 1**). Among these predicted protein-coding genes, 24,049 (75.95%) genes from the Female V1 genome and 27,284 (71.8%) genes from the Male V2 genome were functionally annotated in the GO, KEGG, EggNOG, Pfam and UniProt databases (**Supplementary Table 3**). In addition, 2,488 and 2,910 noncoding RNAs were separately annotated in the two newly assembled genomes. The Female V1 genome contained 976 tRNAs, 178 rRNAs, 1,141 snRNAs and 193 miRNAs. Similarly, four types of noncoding RNAs were identified in the Male V2 genome, including 1,216 tRNAs, 214 rRNAs, 1,253 snRNAs and 182 miRNAs (**Supplementary Figures 4**, **5** and **Supplementary Table 4**).

### Whole-genome duplication analysis

WGD analysis was performed by calculating synonymous substitutions per synonymous site (Ks) values in the two newly assembled genomes. The density distribution of Ks values exhibited two peaks both in Female V1 and Male V2, with Ks values of 0.45 and 2.29 (**Figure 3B**). After the early γ duplication event that affected eudicots, *E. ulmoides* experienced a more recent WGD event approximately 27.3 Mya. The collinearity analysis of male and female revealed a 1:1 synteny pattern (**Supplementary Figure 6**). The divergence time between female and male *E. ulmoides* was defined with a Ks peak value of 0.075 (**Figure 3B**).

### MADS-box genes involved in sex differentiation

In the Female V1 and Male V2 genome, 76 and 81 MADS-box genes were separately identified, and further phylogenetic analysis of these genes was performed (**Supplementary Figure 7**). Based on the transcriptome analysis, 30 MADS-box genes of Female V1 were differentially expressed at different developmental stages of male and female flower buds. In addition, nine genes involved in the ABCDE model of floral development were identified in these MADS-box genes, which included three A-class genes (*EuAP1, EuFUL1, EuAGL6*), one B-class gene (*EuAP3*), one C-class gene (*EuAG*), two D-class genes (*EuSHP1, EuSHP2*), and two genes (*EuSEP1, EuSEP2*) that were associated with the E function (**Supplementary Table 5**). Nine MADS-box genes involved in the ABCDE model of floral development were identified in Male V2. We found high homology between male and female lineages (**Supplementary Figure 8**) and the gene structure and domain sequence of these genes were similar (**Supplementary Figures 9**, **10**). In addition, we compared the obtained sex differentiation genes with the related sex differentiation genes in *Nymphaea colorata*, and the genes involved in sex differentiation in *E. ulmoides* and *N. colorata* showed high homology (**Supplementary Figure 11**).

**Figure 5:**
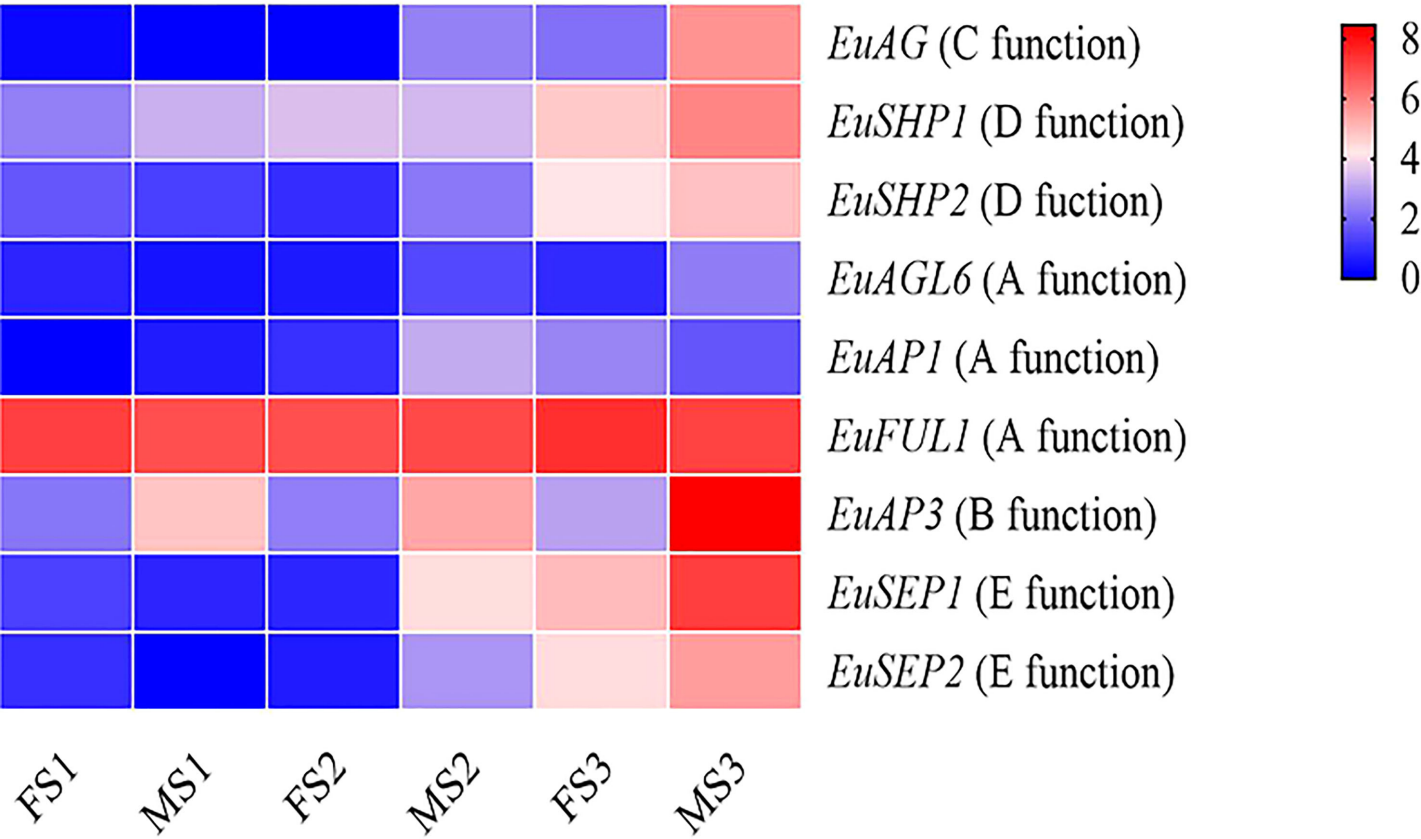
Heatmap of sex determination genes expression data in different stages of flower bud development in Female and male *E. ulmoides*. FS1: female floral organ induction stage flower bud, MS1: male floral organ induction stage flower bud, FS2: female floral organ morphological differentiation initial stage flower bud, MS2: and male floral organ morphological differentiation initial stage flower bud, FS3: female floral organ maturity stage flower bud, MS3: male floral organ maturity stage flower bud. Various color blocks represent the normalized gene expression levels of candidate genes involved in sex determination at different stages of flower bud development in Female and male *E. ulmoides*. The six boxes in one row of each heatmap (left to right) correspond to the expression levels in FS1, MS1, FS2, MS2, FS3, and MS3. Each row in the heatmap corresponds to one gene.

The expression levels of the ABCDE genes at the floral organ maturity stage were higher than those in the floral organ induction stage and morphological differentiation initial stage. Except for the *EuFUL1* and *EuSHP2* genes, the other seven ABCDE genes were differentially expressed between the male and female flower buds. Seven ABCDE genes were differentially expressed during the floral organ maturity stage, with four differentially expressed genes in the floral organ morphological differentiation initial stage, and only one gene differentially expressed in the floral organ induction stage. The *EuAP3* gene was differentially expressed in three different developmental stages of male and female flower buds (**Figure 5** and **Supplementary Figure 12**).

### Identification of the genes involved in α-linolenic acid synthesis

According to the functional annotation, 12 genes encoding key enzymes involved in the main synthesis and metabolic pathways of linolenic acid were identified in the Female V1 genome. Among them, six genes of four key enzymes related to the synthesis of α-linolenic acid were identified, including two acyl-ACP desaturase (FAB2) genes—*EU0119133* and *EU0120166*, which are homologues of SAD and perform the same function of converting stearoyl-ACP (C18:0-ACP) to oleoyl-ACP (C18:1-ACP) (Kazaz et al., 2020); one fatty acyl-ACP thioesterase A (FATA) gene*—EU0103200;* two omega-6 fatty acid desaturase (FAD2, FAD6) genes—*EU0105412* and *EU0128492;* and one omega-3 fatty acid desaturase (FAD7) gene—*EU0103017*. Six key enzyme genes for α-linolenic acid metabolism were also identified, including four lipoxygenase (LOX) genes—*EU0114412, EU0114414, EU0119724*, and *EU0119725*—and two alpha-dioxygenase (DOX) genes, *EU0107025, EU0131326* (**Supplementary Table 6**).

**Figure 6:**
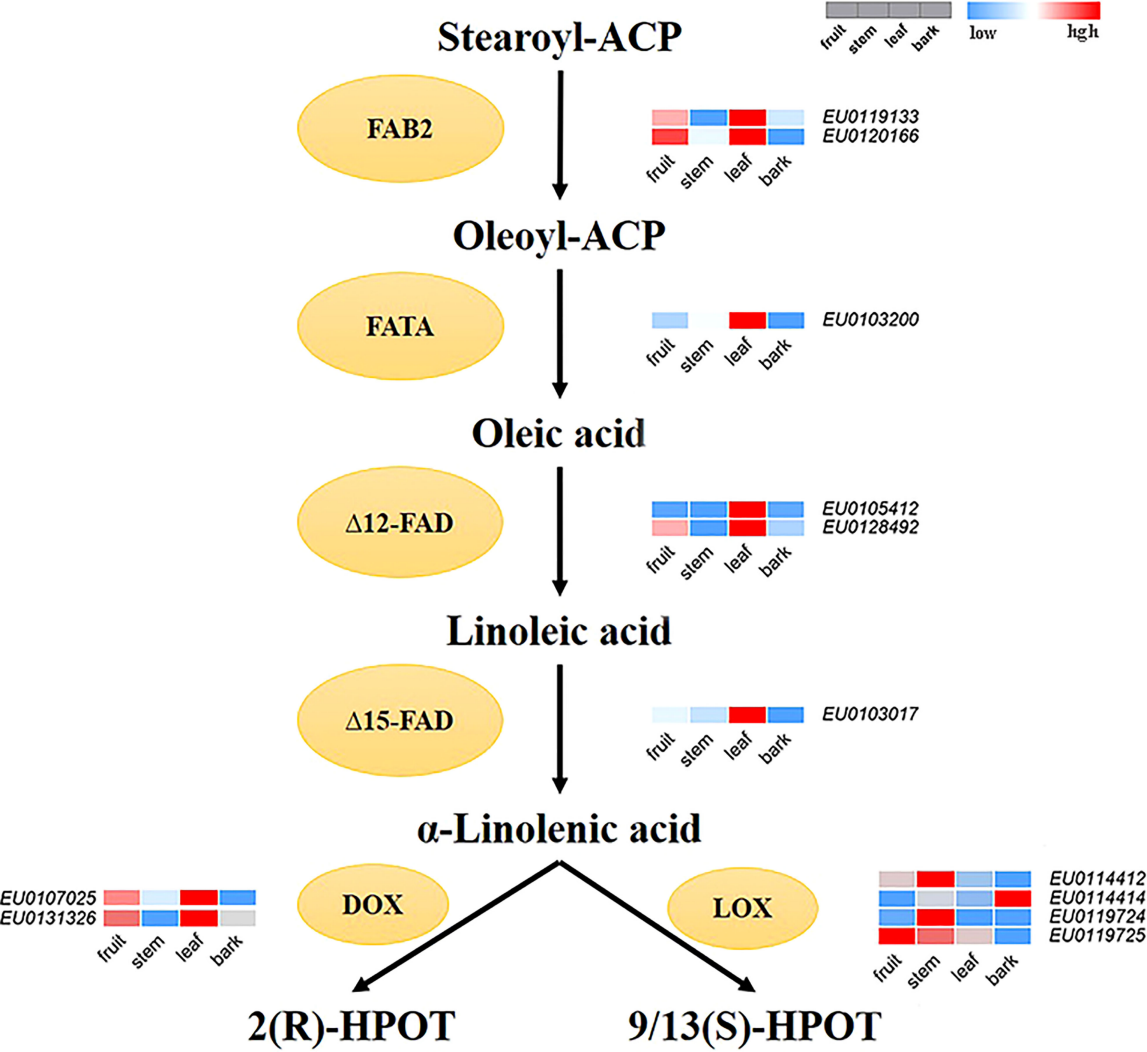
The reconstructed pathway of α-linolenic acid biosynthesis and metabolism in *E. ulmoides*. FAB2, Aacyl-ACP desaturase; FATA, acyl-ACP thioesterase; Δ12-FAD, omega-6 fatty acid desaturase; Δ15-FAD, omega-3 fatty acid desaturase; LOX, lipoxygenase; DOX, alpha-dioxygenase. Various color blocks represent the normalized gene expression levels of candidate genes related to α-linolenic acid biosynthesis and metabolism in *E. ulmoides*. The four boxes in one row of each heatmap (left to right) correspond to the expression levels in fruit, stem, leaf, and bark. Each row in the heatmap corresponds to one gene.

In addition, the expression levels of 12 identified key enzyme genes were compared between four parts of the fruit, stem, bark, and leaf. A heatmap of gene expression levels was also drawn (**Figure 6**). The expression levels of α-linolenic acid synthesis-related genes were higher in fruits and leaves than that in bark and stems. Overall, the expression levels of α-linolenic acid synthesis-related genes were much higher than those of metabolism-related genes. In particularly, the gene *EU0103017*—encoding FAD7, a key enzyme for linolenic acid synthesis—was highly expressed.

## Discussion

*E. ulmoides* is an important economic plant because its bark, leaf, flower, fruit, and other tissues are rich in active secondary metabolites with important medicinal and economic value (Wang et al., 2019). High-quality *E. ulmoides* genomes have been useful for exploring the mechanisms of sex differentiation, synthesis and metabolism of important compounds. We assembled high-quality female and male genomes using PacBio and Hi-C technologies. There were 31,665 protein coding genes identified in Female V1 as compared to the 37,998 predicted genes in Male V2, and the repetitive sequence size in Female V1 was higher than that in Male V2. These results indicated that some differences in the genomes of male and female *E. ulmoides* were produced during the evolutionary process. Compared to previously published genomes (Wuyun et al., 2018) (Male V1), the quality of the reassembled version in this study (Male V2) was significantly increased. For example, superscaffold N50 was 25 times higher in Male V2 as compared to Male V1, and there were 1,266 new protein coding genes in addition to the 26,732 identified in Male V1. All these results indicated that there was greater accuracy and completeness in the Male V2 genome assembly as compared to the Male V1 genome. These two newly genomes provide a valuable resource for understanding sex differentiation and synthesis of important compounds in *E. ulmoides*, and they can be widely applied to genetics and genomics studies, such as the development of markers related to sex identification, detection of genes that synthesize important substances, and studies on the evolutionary history of *E. ulmoides*.

TEs is an important driving force to promote genome evolution, and the most important driving force is the accumulation of LTR-RTs (Sahebi et al., 2018; Lanciano and Cristofari, 2020). Further analysis showed that LTR-RTs erupted approximately 2 Mya, and cold tolerance may explain why *E. ulmoides* survived the Quaternary glaciation (Cros et al., 2020) approximately 2 Mya. Another insertion peak was identified in the Male V2 genome, this may be related to a greater number of repetitive sequences in the male genome. Whole genome duplication (WGD), also known as a paleopolyploidy event, provides abundant evolutionary materials for species and is considered as an important driving force for the formation and evolution of species (Soltis and Soltis, 2016; Clark and Donoghue, 2018). Most plants experience WGD, and many WGD events in angiosperms were detected during the Cretaceous-Tertiary period (145.5–65.5 Mya) (Vanneste et al., 2014). There is an obvious peak in the Ks distribution at the Ks value of 2.29375 in the two genomes, which indicated that males and females underwent a WGD event approximately 139 Mya (γ-WGT, 122–164 Mya) (Salse, 2016). Plants undergoing WGD events can enhance their environmental adaptability and stress resistance, thus promoting species evolution (del Pozo and Ramirez-Parra, 2015). Therefore, γ-WGT event may increase the adaptability of *E. ulmoides* to harsh environments, thus enabling *E. ulmoides* to resist the extinction event that occurred in the Cretaceous-Tertiary period. The peak at 0.45 in male genome is not as obvious as that in female, which might be the comparatively smaller continuity (Contig N50 17.06 kb) and lower quality of male genome than that of female genome. To detect whether there is a WGD event here, we have performed collinearity analysis. The 1:1 synteny pattern between male and female genomes also revealed that they have undergone the same number of WGD events. Besides, the divergence time between female and male *E. ulmoides* was identified with a Ks peak value of 0.075, while the recent WGD, specifically occurred in female, was with the Ks peak value of 0.45, which indicated that the recent WGD occurred before their differentiation.

There were nine MADS-box genes involved in the ABCDE mode in both female and male *E. ulmoides*, and their gene structure and domain sequence were conservative, suggesting their important role in floral development. During the development of male and female *E. ulmoides* flower buds, the greatest number of MADS-box differentially expressed genes was observed in the floral organ maturity stage, followed by the floral organ morphological differentiation initial stage, and the same was true for ABCDE genes. These results showed that the male and female flower buds of *E. ulmoides* were identical in the early developmental stage. However, with the development of female and male flower buds, the morphological differences gradually became obvious, and the differential genes increased. The ABCDE model of floral organ has been studied in *Nymphaea colorata* (Zhang et al., 2020), and the genes involved in floral organ in *E. ulmoides* and *N. colorata* showed high homology, which indicated that the genes related to flower development identified in *E. ulmoides* were reliable. In the ABCDE flower development model, B, C, and E genes jointly control stamen development (Hu et al., 2021). During the floral organ maturity stage of *E. ulmoides*, a large number of MADS-box family genes were differentially expressed in male and female flower buds. In particular, we observed that the B-class gene *EuAP3* was differentially expressed during the three stages of male and female flower bud development, and the expression level in male flower buds was significantly higher than that in female flower buds, which further supports the role of *EuAP3* in regulating stamen development. The expression of the C-class gene (*EuAG*) was also upregulated in male flower buds. This indicated that similar to the AG gene function in *Arabidopsis* and rice, the *EuAG* gene was involved in the development of stamens. *EuAP3* and *EuAG* are the key regulatory genes for stamen development and may be candidate genes for regulating sex differentiation.

*E. ulmoides* is rich in α-linolenic acid, which is synthesized from stearic acid under the catalysis of a series of fatty acid desaturases (Liu et al., 2020). First, stearoyl-ACP (C18:0-ACP) is catalyzed by enzyme FAB2 to produce oleoyl-ACP (C18:1-ACP), which is then converted to oleic acid (OA, C18:1) by the FATA enzyme. Oleic acid is desaturated by the enzyme fatty acid desaturase 2 (FAD2) and fatty acid desaturase 6 (FAD6) to generate linoleic acid (LA, C18:2). The fatty acid desaturase 7 (FAD7) enzyme catalyzes the synthesis of α-linolenic acid (ALA, C18:3) from LA in *E. ulmoides.* DOX and LOX are two key enzymes that catalyze the first step of α-linolenic acid metabolism, and they catalyze α-linolenic acid to produce 2(R)-HPOT and 9/13(S)-HPOT, respectively (Gfeller et al., 2010; Shimada and Hara-Nishimura, 2015).

Transcriptome analysis results showed that the expression of synthesis-related genes was high, and there was very high expression of the genes encoding the key enzyme ω-3 fatty acid desaturase (FAD7), which is involved in α-linolenic acid synthesis and allows *Eucommia* to synthesize sufficient α-linolenic acid. The expression of metabolism-related genes was lower or they were not expressed, and thus, α-linolenic acid could be efficiently accumulated in *E. ulmoides*. These results may explain why the α-linolenic acid content in *E. ulmoides* is higher than that in other plants. In addition, the expression level of genes related to α-linolenic acid synthesis in fruit and leaf was higher than that in bark and stem. Although the expression of genes related to α-linolenic acid synthesis was higher in leaf (*EU0119133, EU0120166, EU0103017*), the expression of genes related to the downstream α-linolenic acid metabolic pathway was also higher in leaf (*EU0107025, EU0131326*). This allows the synthesis of α-linolenic acid in the leaf to be greatly metabolized, while the α-linolenic acid in the fruit can be accumulated, which explains why the α-linolenic acid is mainly distributed in the fruit of *E. ulmoides*.

In the current study, two high-quality *E. ulmoides* genomes are provided, which are helpful for evolutionary and genomics research. Using these genomic data, we identified WGD events and evaluated the LTR outbreak time to provide a basis for future studies on genomic evolution and stress resistance of *E. ulmoides*. MADS-box family genes were also identified, and the genes related to flower development in *E. ulmoides* were predicted according to the expression patterns of genes in male and female flower buds, and finally the key candidate genes that regulate sex differentiation were identified. In addition, the key regulatory genes for α-linolenic acid synthesis and metabolism were identified, and the mechanism of α-linolenic acid accumulation was revealed. According to these genes, *E. ulmoides* can be improved by directed genetic modification. Overall, our research results not only provide valuable information for the evolutionary analysis of *E. ulmoides* but also advance our understanding of the molecular mechanism of sex differentiation and α-linolenic acid accumulation. This study will accelerate the breeding of superior varieties and enable us to more thoroughly explore and use the value inherent in *E. ulmoides*.

## Data availability statement

The datasets presented in this study can be found in online repositories. The names of the repository/repositories and accession number(s) can be found below: https://www.ncbi.nlm.nih.gov/, PRJNA792509.

## Author contributions

HD and LW conceived this study; QD, ZW, and PL worked on the data analysis and wrote the manuscript; JQ, FH, LD, ZhS LZ, and HZ contributed to the sample preparation; ZoS provided sequencing service; LY and LW revised the manuscript. All authors contributed to the article and approved the submitted version.
