# Supplementary material for: The chromosome-level genome of Eucommia ulmoides provides insights into sex differentiation and α-linolenic acid biosynthesis
Source: Front Plant Sci. 2023 Mar 31;14:1118363. doi: 10.3389/fpls.2023.1118363 (PMC10102601; doi:10.3389/fpls.2023.1118363)
Supplement: Supplementary file 1 [file DataSheet_1.docx]

**SUPPORTING INFORMATION**

Qingxin Du^1,2,3†^, Zixian Wu^4†^, Panfeng Liu^1,2,3†^, Jun Qing^1,2,3^, Feng He^1,2,3^, Lanying Du^1,2,3^, Zhiqiang Sun^1,2,3^, Lili Zhu^5^, Hongchu Zheng^6^, Zongyi Sun^7^, Long Yang^4*^, Lu Wang^1,2,3*^, Hongyan Du^1,2,3*^

^1^ Research Institute of Non-timber Forestry, Chinese Academy of Forestry, Zhengzhou 450003, China.

^2^ Key Laboratory of Non-timber Forest Germplasm Enhancement & Utilization of National Forestry and Grassland Administration, Chinese Academy of Forestry, Zhengzhou 450003, China.

^3^ Engineering Research Center of Eucommia ulmoides, State Forestry and Grassland Administration, Zhengzhou 450003, China.

^4^ Agricultural Big-Data Research Center and College of Plant Protection, Shandong Agricultural University, Taian 271018, China.

^5^ Academy of Chinese Medical Sciences, Henan University of Chinese Medicine, Zhengzhou 450046, China.

^6^ Product Departmeng, Henan Jinduzhong Agricultural Science and Technology Co., Ltd., Yanling 461200, China.

^7^ Operation Department, Grandomics Biosciences Co., Ltd., Wuhan 430076, China.

**The Chromosome-level genome of *Eucommia ulmoides* provides insights into sex differentiation and α-linolenic acid biosynthesis**

**Supplementary Figures**


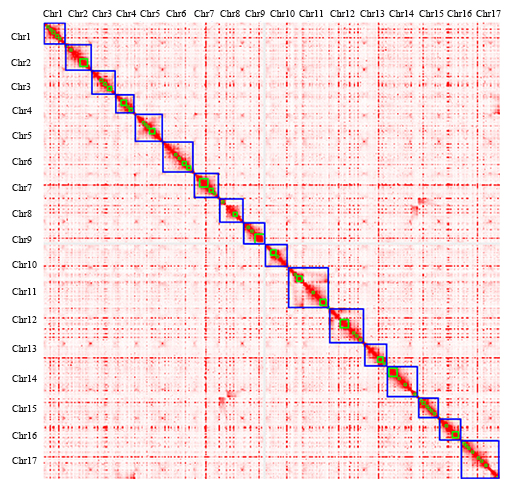


**Supplementary Fig. 1 Genome-wide analysis of chromatin interactions in Female V1 genome.** The x-axis and y-axis stand for the chromosomes of assembled genome, the blue blocks indicate the chromosome boundary, the color depth implies the interact intensity.


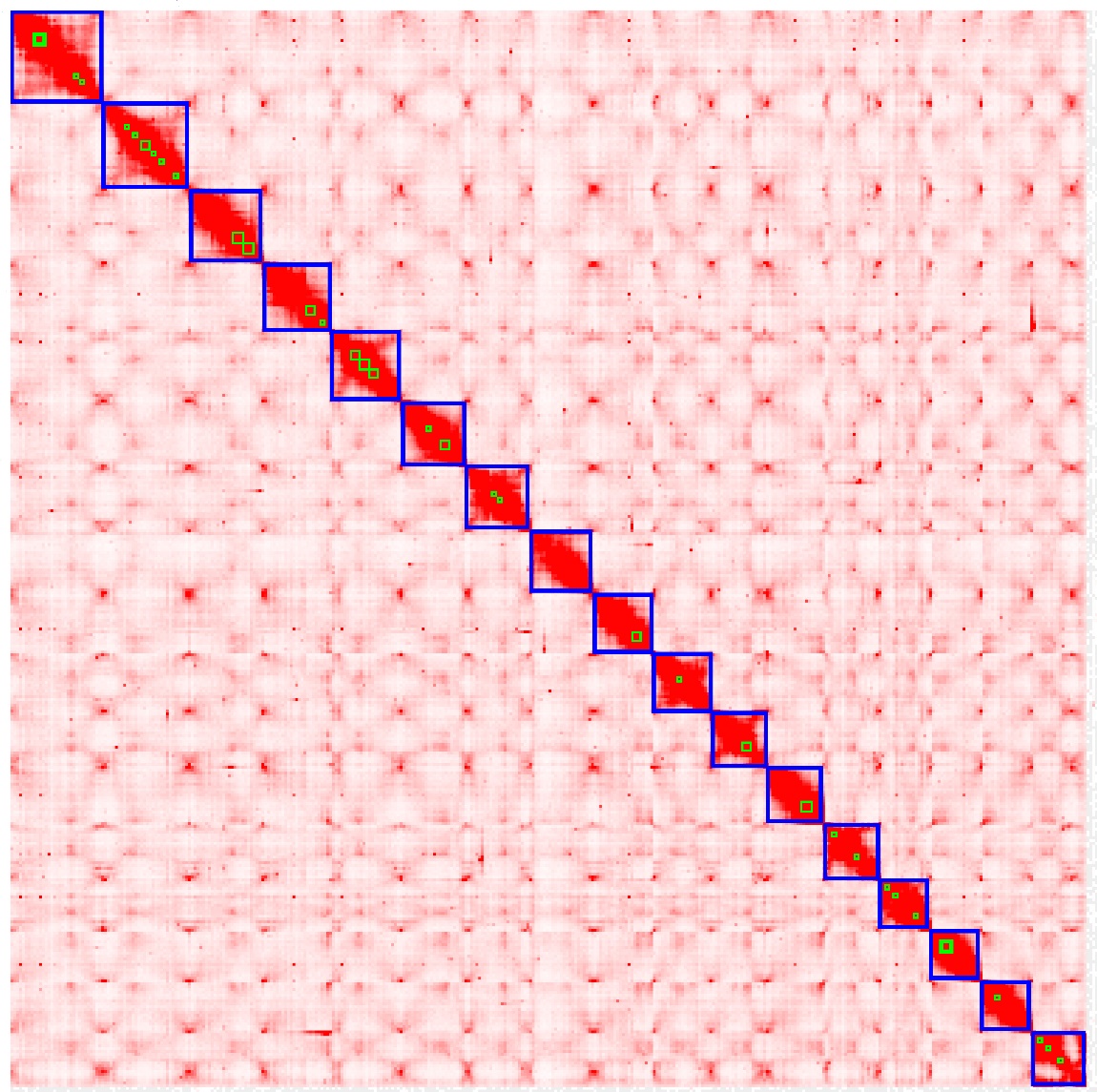


**Supplementary Fig. 2 Genome-wide analysis of chromatin interactions in Male V2 genome.** The x-axis and y-axis stand for the scaffolds of assembled genome, the blue blocks indicate the scaffold boundary, the color depth implies the interact intensity.

**Supplementary Fig. 3 BUSCO assessment of the *E. ulmoides* genome.**


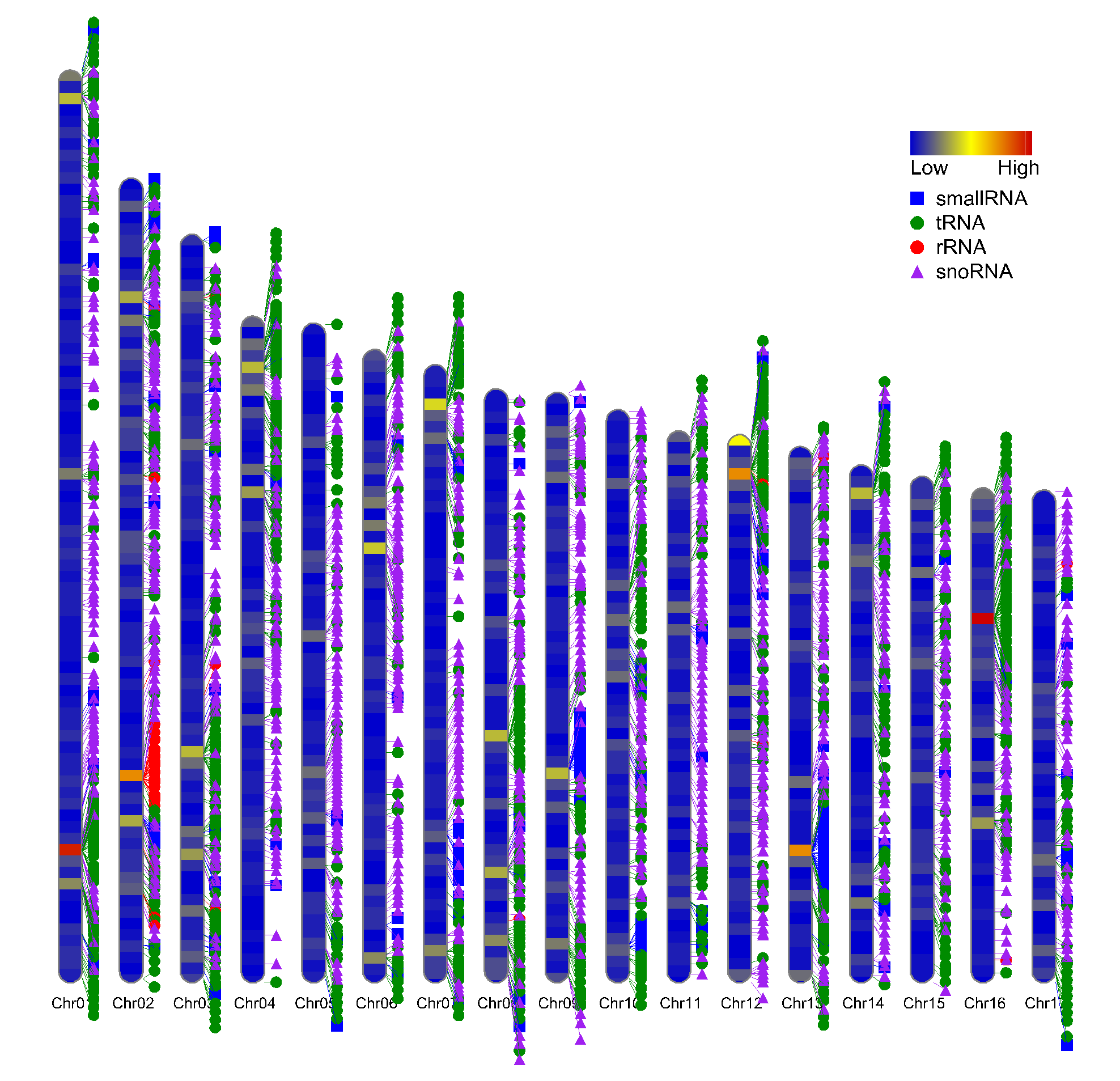


**Supplementary Fig. 4 Non-coding RNA genes in the genome of Female V1 genome.**


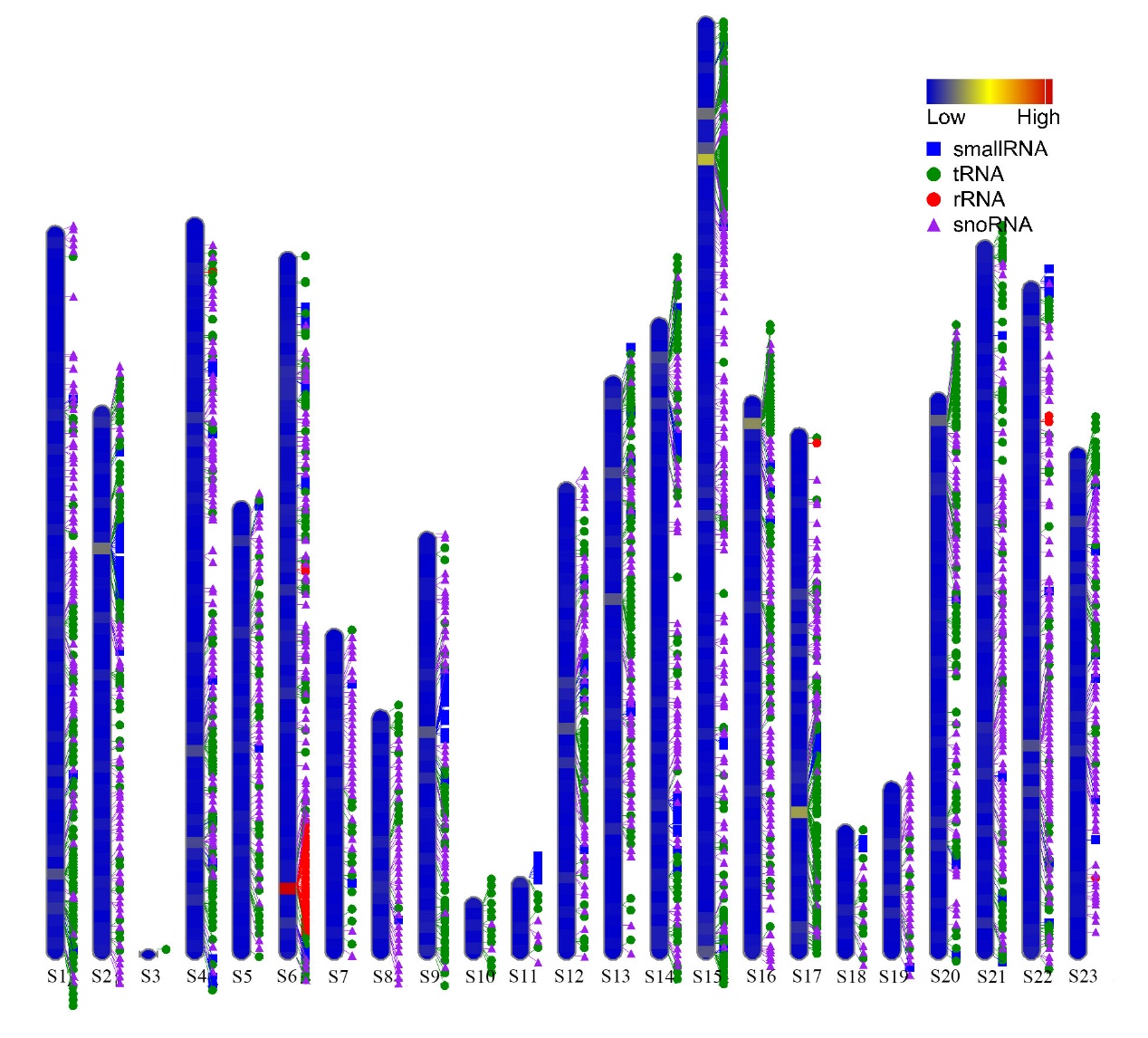


**Supplementary Fig. 5 Non-coding RNA genes in the genome of Male V2 genome.**


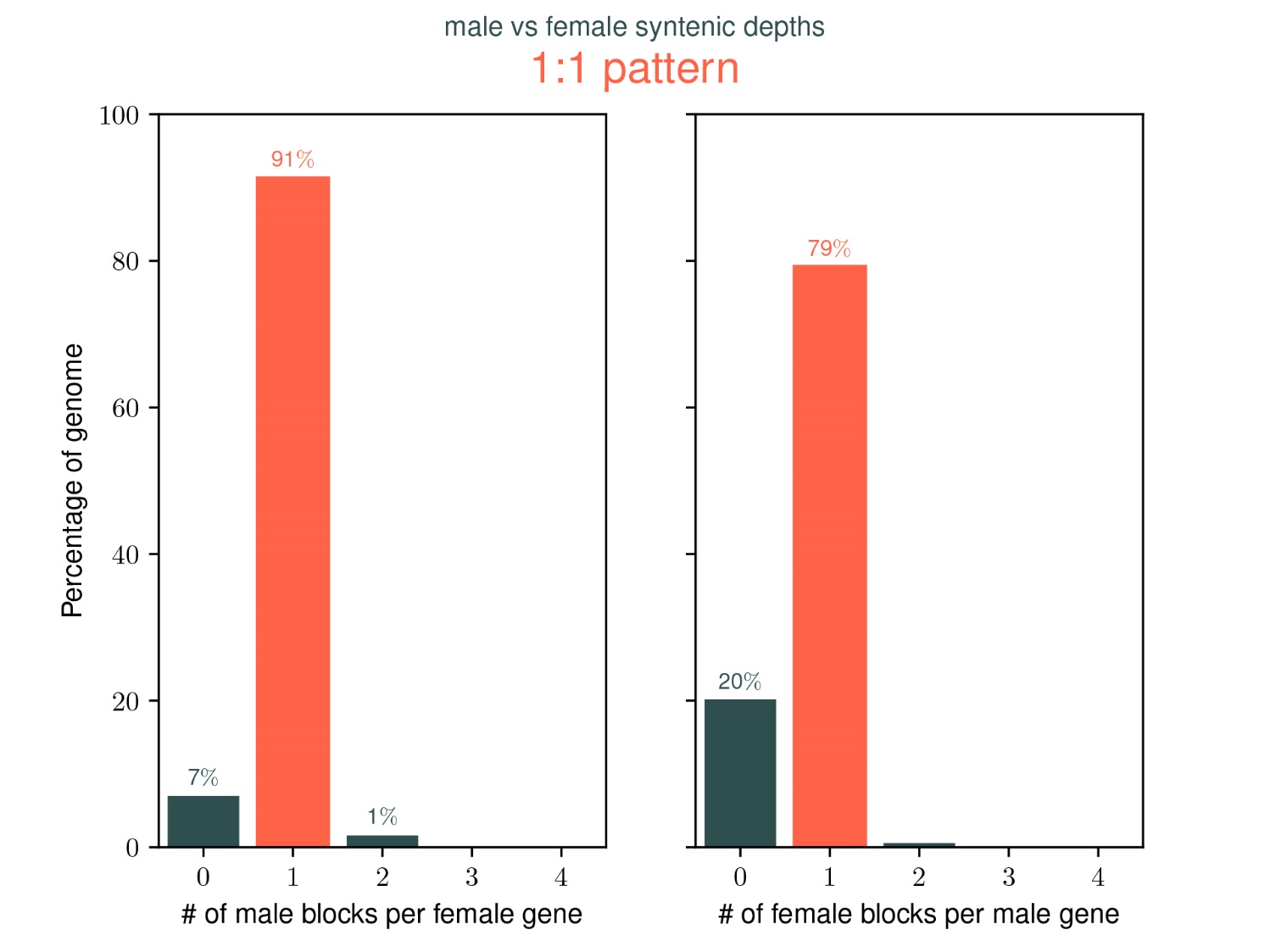


**Supplementary Fig. 6** Synteny pattern of female and male *E. ulmoides*.


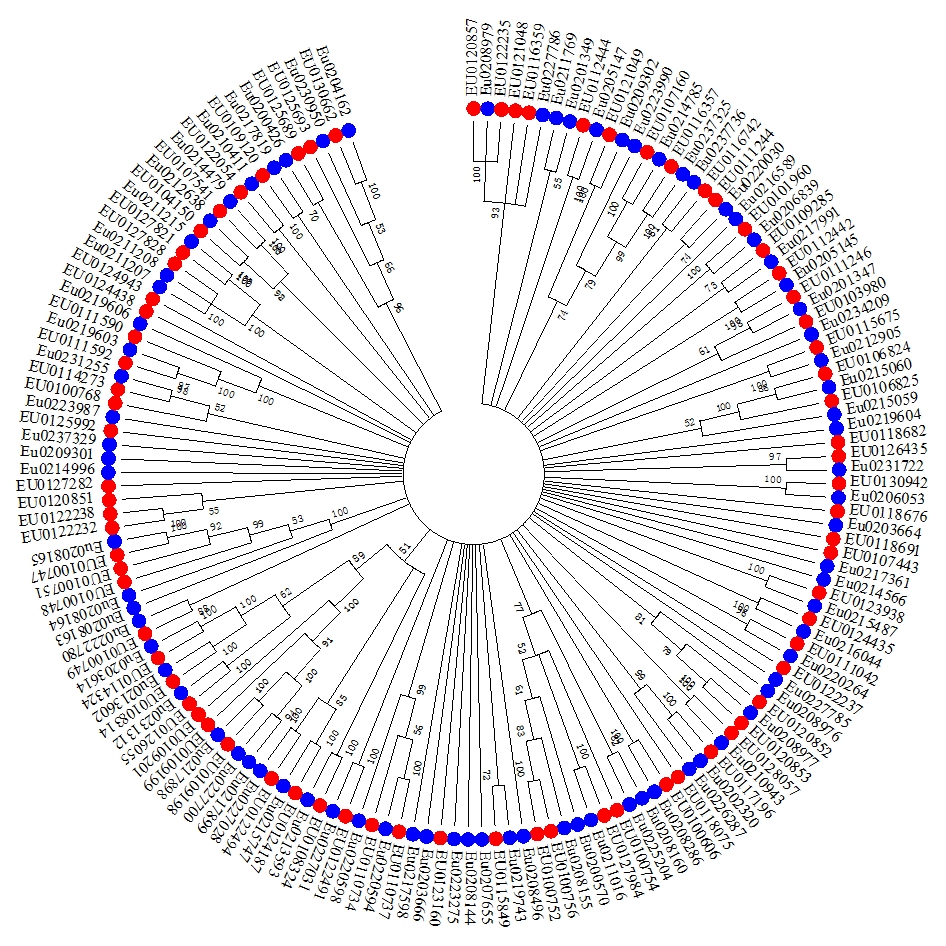


**Supplementary Fig. 7** **Phylogenetic analysis of the** **MADS-box genes from Female V1 and Male V2.** The red dots represent the MADS-box genes in Female V1 genome, blue dots represent the MADS-box genes in Male V2 genome.

**
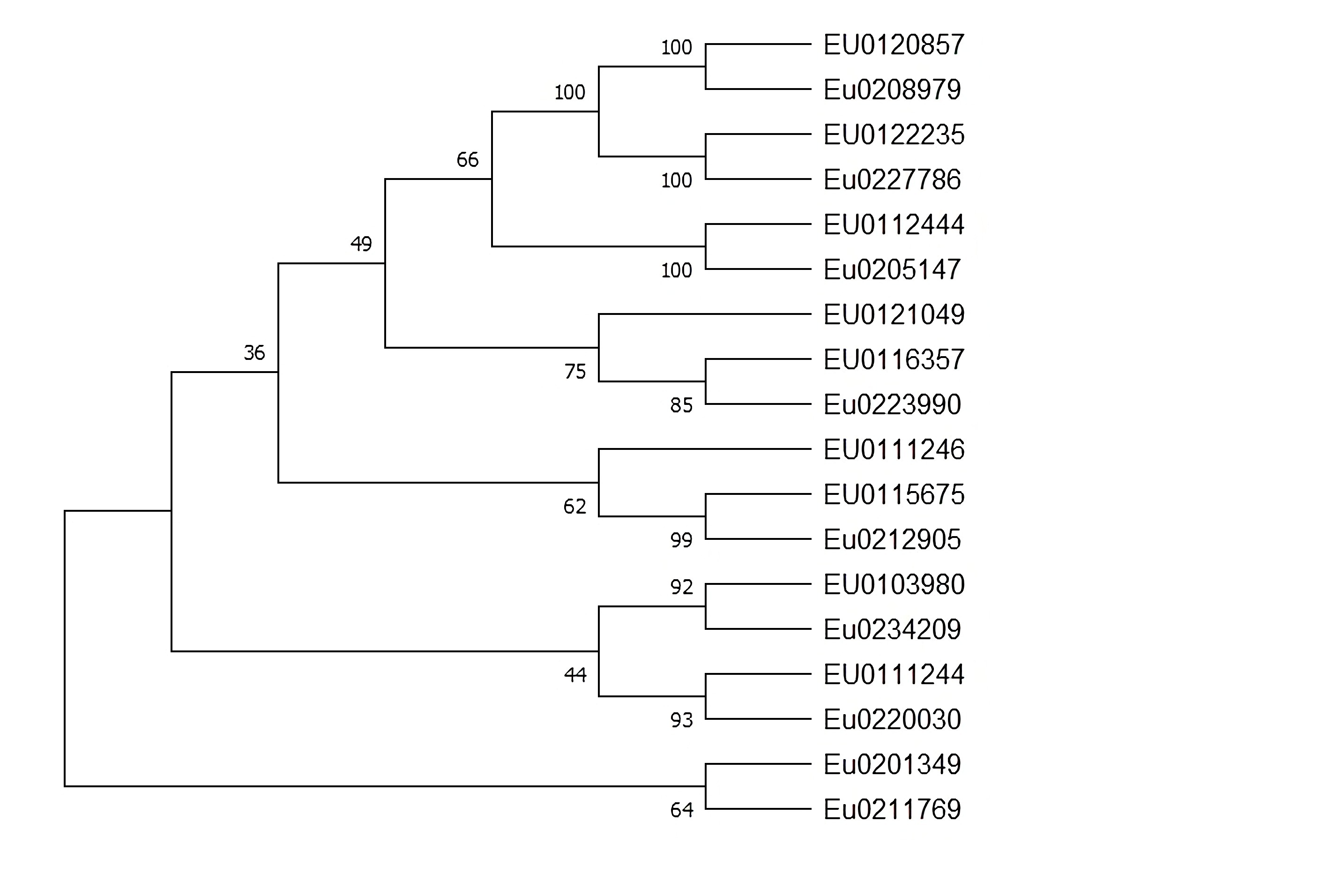
**

**Supplementary Fig. 8 Phylogenetic analysis of the MADS-box genes involved in the ABCDE model of floral development from Female V1 and Male V2.**

**
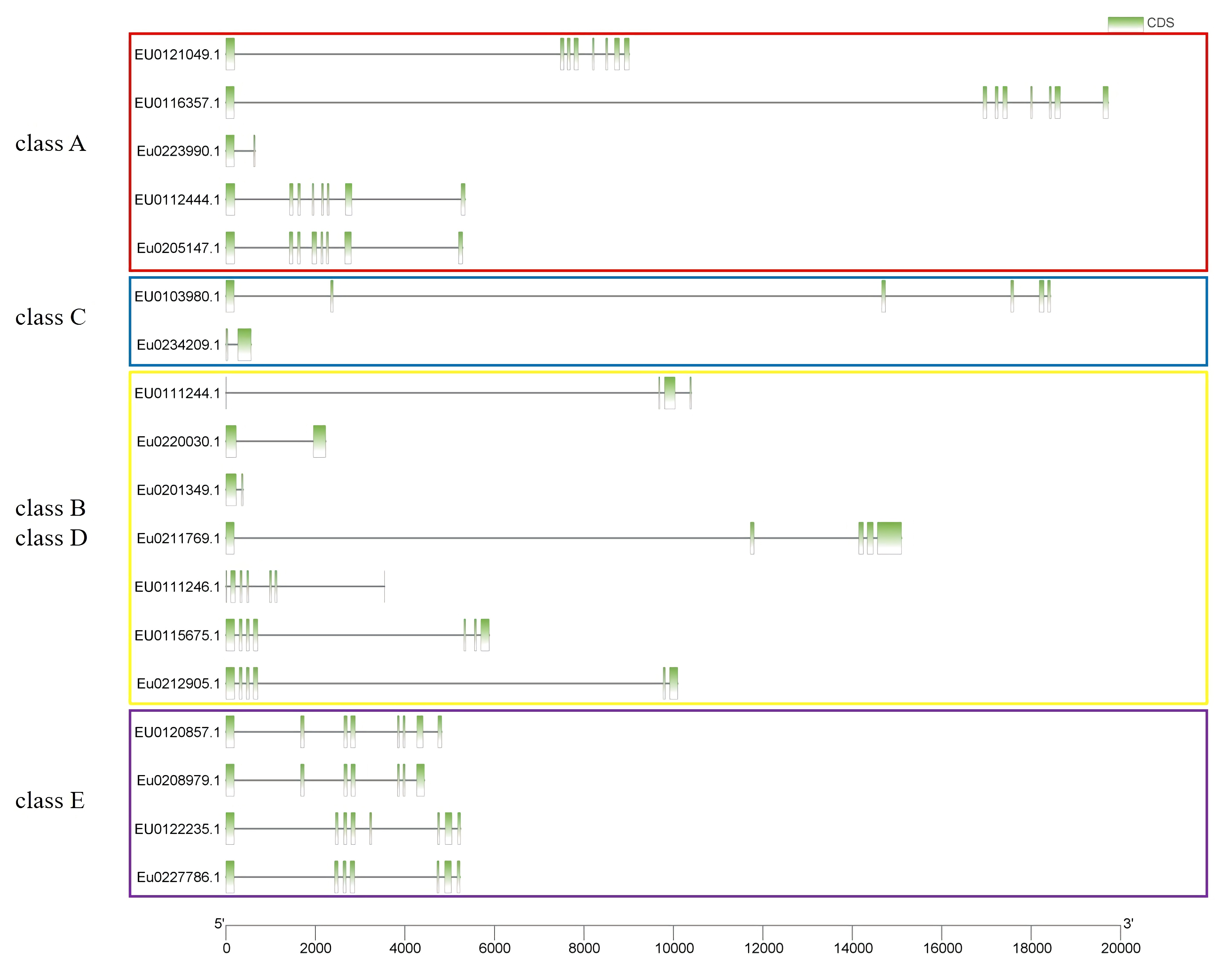
**

**Supplementary Fig. 9 The gene structure of the MADS-box genes involved in the ABCDE model of floral development from Female V1 and Male V2.**

**
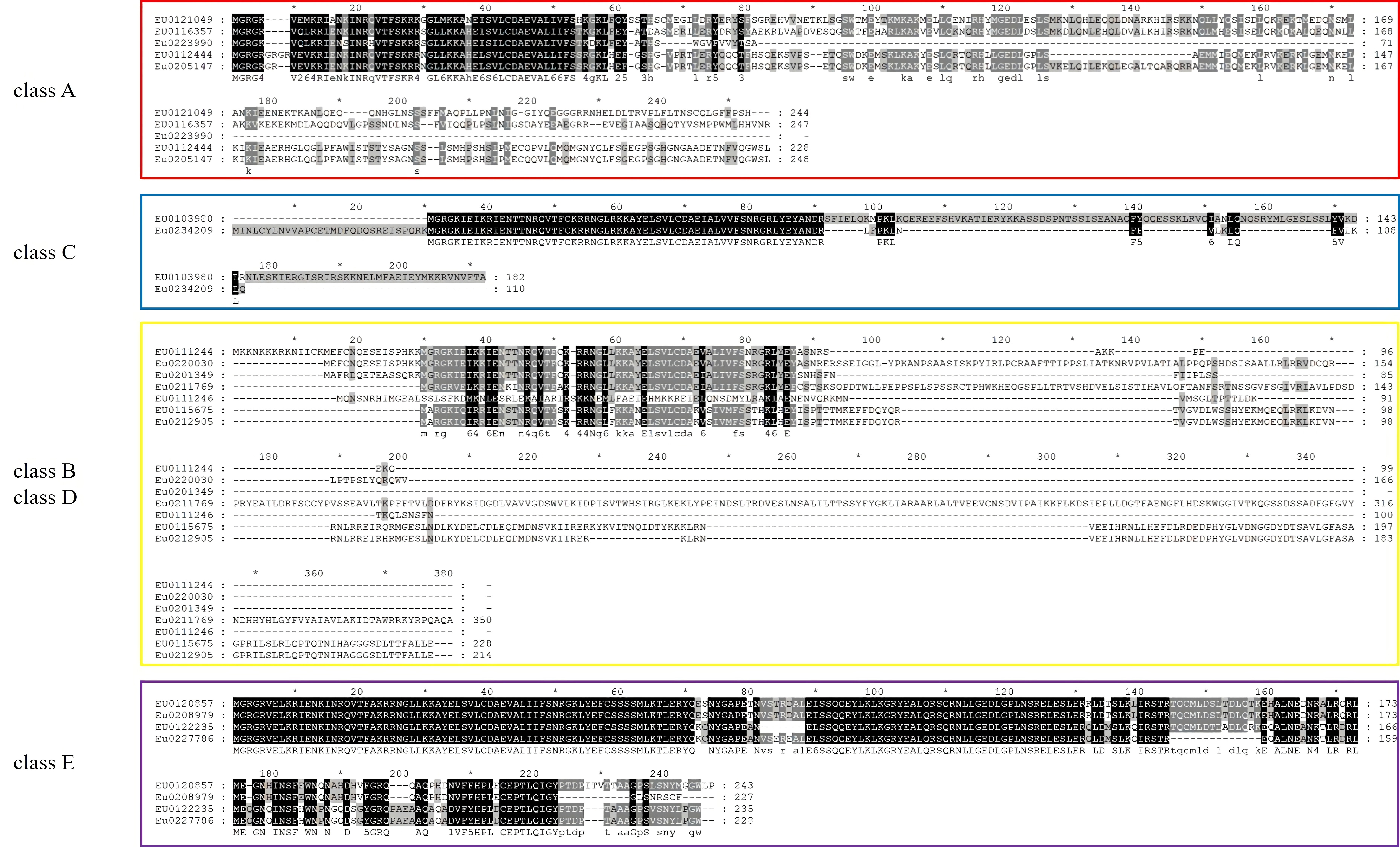
**

**Supplementary Fig. 10 The genomic sequence of the MADS-box genes involved in the ABCDE model of floral development from Female V1 and Male V2.**

**
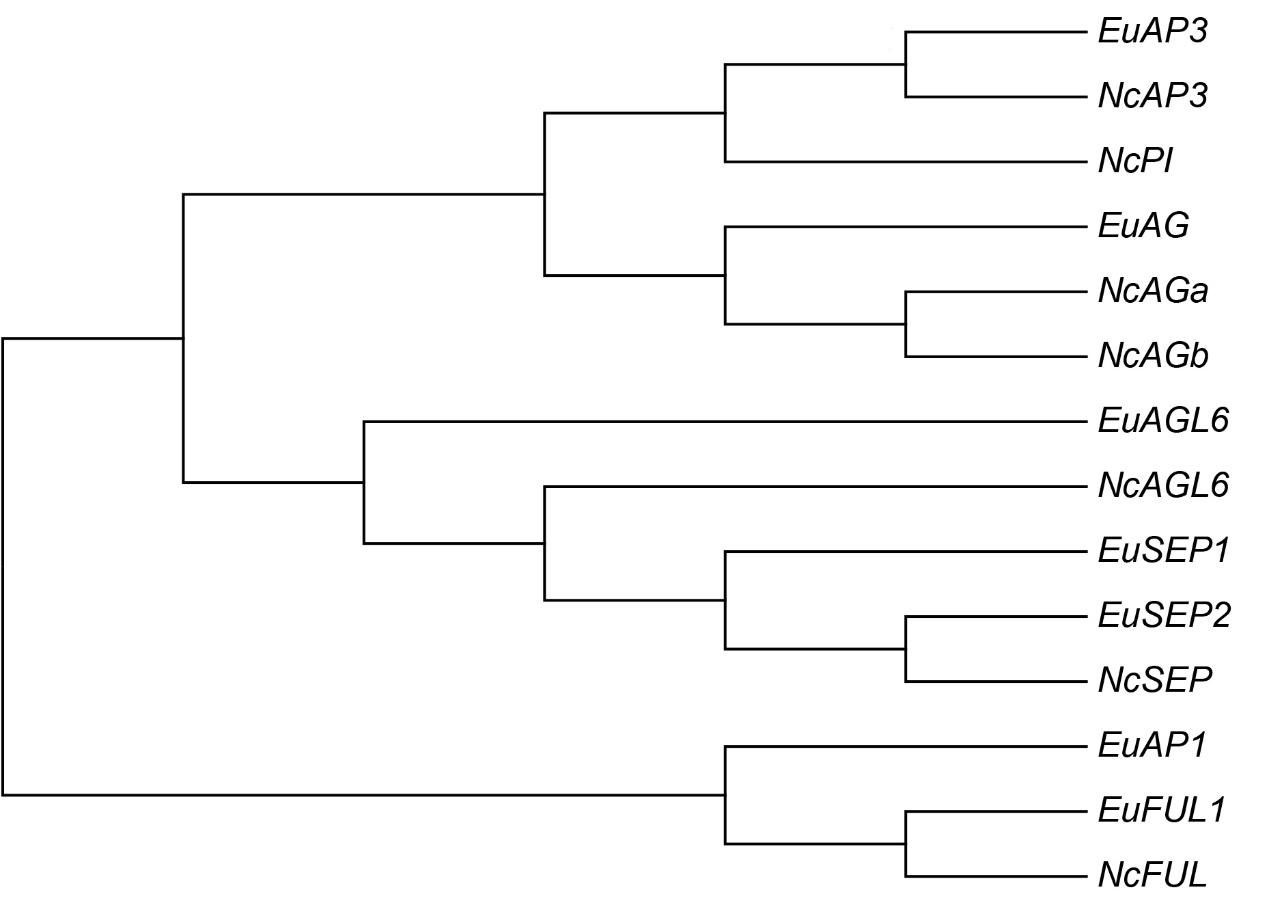
**

**Supplementary Fig. 11** **Phylogenetic analysis of the** **genes related to sex differentiation in *E. ulmoides* and *N. colorata*.**


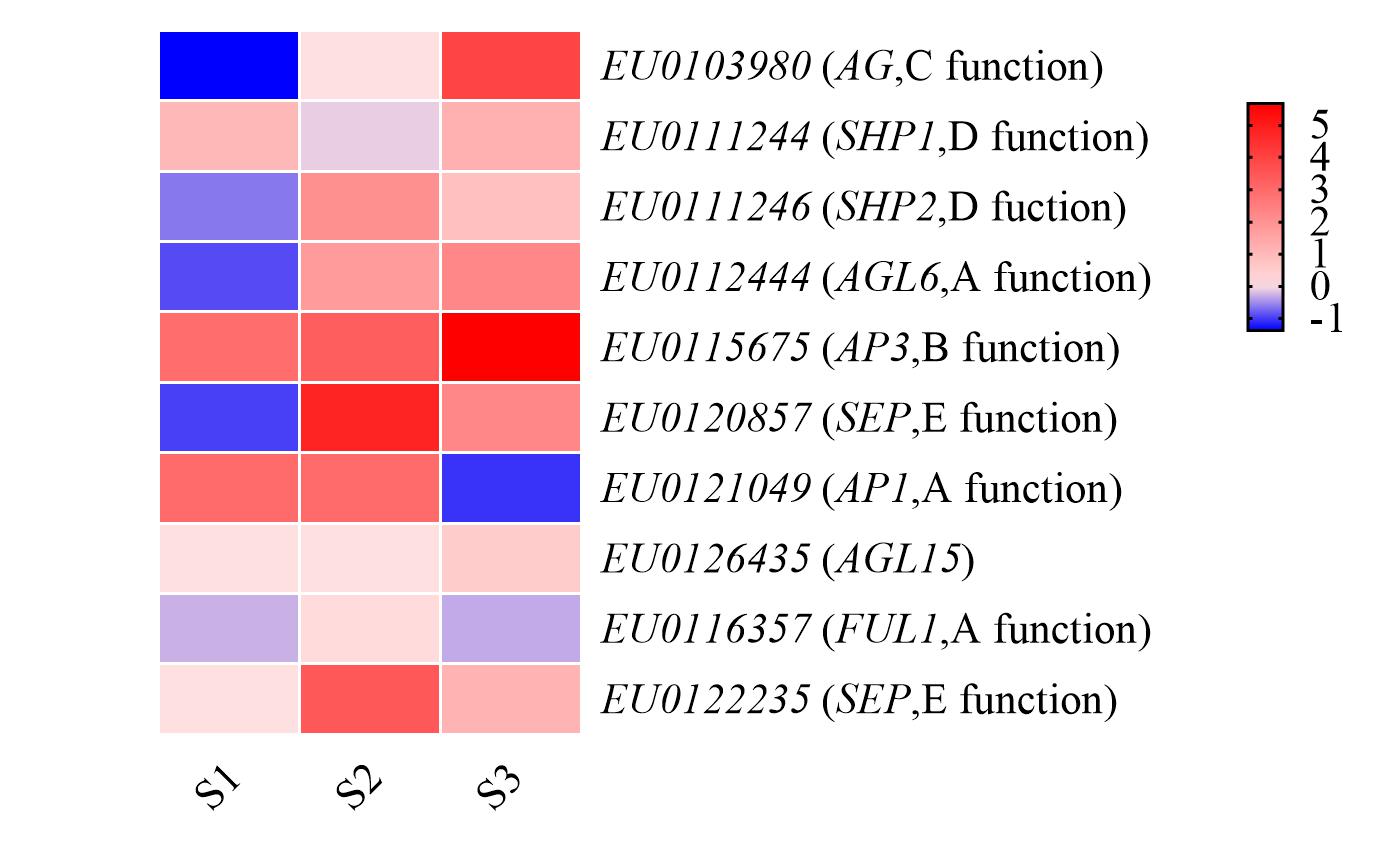


**Supplementary Fig. 12 The expression changed the level log_2_(fold-change) of sex determination genes in different stages of flower bud development in female and male *E. ulmoides.*** S1: floral organ induction stage flower bud, S2: floral organ morphological differentiation initial stage flower bud, S3: flower organ maturity stage flower bud.

**Supplementary Tables**

**Supplementary Table 1** **BUSCO assessment of the *E. ulmoides* genome.**

|  | **Complete and single-copy BUSCOs (S) %** | **Complete and duplicated BUSCOs (D) %** | **Fragmented BUSCOs (F) %** | **Missing BUSCOs (M) %** | **Complete BUSCOs (C) %** |
| --- | --- | --- | --- | --- | --- |
| **Male V1** | 86 | 4 | 2.8 | 7.2 | 90 |
| **Male V2** | 88 | 4.1 | 3.4 | 4.5 | 92.1 |
| **Female V1** | 87.5 | 5.7 | 2.3 | 4.5 | 93.2 |

**Supplementary Table 2 Statistics of repeat sequence in** ***E. ulmoides* genome.**

|  | **Female V1** | | | **Male V2** | | | |  |
| --- | --- | --- | --- | --- | --- | --- | --- | --- |
|  | **Number** | **Length (bp)** | **% of genome** | | **Number** | **Length (bp)** | **% of genome** | |
| **DNA** | 218,225 | 59,046,848 | 5.85 | | 253,911 | 64,559,855 | 5.21 | |
| **LINE** | 43,900 | 27,685,030 | 2.74 | | 50,875 | 30,090,296 | 2.43 | |
| **SINE** | 22,878 | 3,110,478 | 0.31 | | 26,300 | 3,538,417 | 0.29 | |
| **LTR** | 458,462 | 405,083,159 | 40.15 | | 631,283 | 453,300,131 | 36.60 | |
| **LTR/Copia** | 100,859 | 73,275,051 | 7.26 | | 207,805 | 95,075,831 | 7.68 | |
| **LTR/Gypsy** | 159,057 | 141,712,314 | 14.05 | | 267,564 | 145,166,693 | 11.72 | |
| **Unclassifed** | 662,091 | 196,724,201 | 19.50 | | 825,630 | 221,868,053 | 17.92 | |
| **Total** | 1,405,556 | 691,649,716 | 68.55 | | 1,787,999 | 773,356,752 | 62.45 | |

**Supplementary Table 3 Functional annotation of predicted genes in *E. ulmoides* genome.**

|  | **Female V1** | | **Male V2** | |
| --- | --- | --- | --- | --- |
|  | **Number** | **Percent (%)** | **Number** | **Percent (%)** |
| **Total** | 31,665 | 100.00 | 37,998 | 100.00 |
| **GO** | 13,173 | 41.60 | 14,340 | 37.74 |
| **KEGG Pathway** | 10,881 | 34.36 | 11,864 | 31.22 |
| **EggNOG** | 21,252 | 67.12 | 23,379 | 61.53 |
| **Pfam** | 14,928 | 47.14 | 24,391 | 64.19 |
| **UniProt** | 14,627 | 46.19 | 14,546 | 38.28 |
| **Annotated** | 24,049 | 75.95 | 27,284 | 71.80 |
| **Unannotated** | 7,616 | 24.05 | 10,714 | 28.20 |

**Supplementary Table 4 Non-coding RNA in the genome of *E. ulmoides*.**

|  | **Female V1** | **Male V2** |
| --- | --- | --- |
| **tRNA** | 976 | 1,261 |
| **rRNA** | 178 | 214 |
| **snRNA** | 1,141 | 1,253 |
| **miRNA** | 193 | 182 |

**Supplementary Table 5 Summary of ABCDE model genes in Female V1 genome.**

| **Gene name** | **Gene ID** | **ABCDE class** | **Chromosome location** | | |
| --- | --- | --- | --- | --- | --- |
| *EuAP1* | *EU0121049* | class A | Chr12 | 3087937-3096952 |  |
| *EuFUL1* | *EU0116357* | class A | Chr09 | 39378175-39397896 |  |
| *EuAGL6* | *EU0112444* | class A | Chr07 | 12994273-12999619 |  |
| *EuAP3* | *EU0115675* | class B | Chr09 | 6525322-6531204 |  |
| *EuAG* | *EU0103980* | class C | Chr02 | 54464671-54483103 |  |
| *EuSHP1* | *EU0111244* | class D | Chr06 | 26014230-26024628 |  |
| *EuSHP2* | *EU0111246* | class D | Chr06 | 26069241-26072783 |  |
| *EuSEP1* | *EU0120857* | class E | Chr12 | 588335-593152 |  |
| *EuSEP2* | *EU0122235* | class E | Chr13 | 587309-592551 |  |

**Supplementary Table 6 Summary of α-linolenic acid synthesis genes in Female V1 genome and their homologous genes ID in Male V2.**

| **Gene name** | **Gene ID** | **Chromosome location** | | **homologous gene ID** |
| --- | --- | --- | --- | --- |
| FAB2-1 | *EU0119133* | Chr11 | 1434268-1437513 | *Eu0235835* |
| FAB2-2 | *EU0120166* | Chr11 | 35520755-35534356 | *Eu0234945* |
| FATA | *EU0103200* | Chr02 | 13727472-13732260 | *Eu0233311* |
| FAD2 | *EU0105412* | Chr03 | 30770157-30771296 | *Eu0229239* |
| FAD6 | *EU0128492* | Chr17 | 16962120-16969216 | *Eu0222002* |
| FAD7 | *EU0103017* | Chr02 | 8611367-8614516 | *Eu0233110* |
| DOX-1 | *EU0107025* | Chr04 | 1772879-1790053 | *Eu0214919* |
| DOX-2 | *EU0131326* | Chr04 | 9896-12888 | *Eu0214919* |
| LOX-2 | *EU0114412* | Chr08 | 29250401-29261262 | *Eu0203519* |
| LOX-2 | *EU0114414* | Chr08 | 29414317-29424182 | *Eu0203517* |
| LOX-3 | *EU0119724* | Chr11 | 11927858-11934848 | *Eu0236333* |
| LOX-4 | *EU0119725* | Chr11 | 11975908-11982842 | *Eu0236333* |
